# Low-cost synthesis of small molecule acceptors makes polymer solar cells commercially viable

**DOI:** 10.1038/s41467-022-31389-y

**Published:** 2022-06-27

**Authors:** Hongyuan Fu, Jia Yao, Ming Zhang, Lingwei Xue, Qiuju Zhou, Shangyu Li, Ming Lei, Lei Meng, Zhi-Guo Zhang, Yongfang Li

**Affiliations:** 1 State Key Laboratory of Chemical Resource Engineering, Beijing Advanced Innovation Center for Soft Matter Science and Engineering, Beijing University of Chemical Technology, 100029 Beijing, China; 2 Analysis & Testing Center, Xinyang Normal University, Xinyang, 464000 Henan China; 3 Beijing National Laboratory for Molecular Sciences, CAS Key Laboratory of Organic Solids, Institute of Chemistry, Chinese Academy of Sciences, 100190 Beijing, China

**Keywords:** Solar cells, Solar cells

## Abstract

The acceptor-donor-acceptor (A–D–A) or A–DA’D–A structured small molecule acceptors (SMAs) have triggered substantial progress for polymer solar cells (PSCs). However, the high−cost of the SMAs impedes the commercial viability of such renewable energy, as their synthesis via the classical pyridine-catalyzed Knoevenagel condensation usually suffers from low reaction efficiency and tedious purifying work-up. Herein, we developed a simple and cheap boron trifluoride etherate-catalyzed Knoevenagel condensation for addressing this challenge, and found that the coupling of the aldehyde-terminated D unit and the A-end groups could be quantitatively finished in the presence of acetic anhydride within 15 minutes at room temperature. Compared with the conventional method, the high reaction efficiency of our method is related to the germinal diacetate pathway that is thermodynamically favorable to give the final products. For those high performing SMAs (such as ITIC-4F and Y6), the cost could be reduced by 50% compared with conventional preparation. In addition to the application in PSCs, our synthetic approach provides a facile and low-cost access to a wide range of D–A organic semiconductors for emerging technologies.

## Abstract (web summary)

The high−cost of the acceptor-donor-acceptor structured small molecule acceptors impedes its commercial viability for polymer solar cells. To address this problem, here, the authors developed a simple and cheap boron trifluoride etherate-catalyzed Knoevenagel condensation with high reaction efficiency.

## Introduction

Polymer solar cells (PSCs) have drawn extensive research attentions in recent years, due to their advantages of light weight, flexibility, low-cost and large-area solution processing^1–4^. Recently, the power conversion efficiencies (PCEs) of PSCs have reached over 18%^5–11^,which is mainly benefited from the emerging narrow-bandgap small molecule acceptors (SMAs) with attractive advantages of easily adjustable structures, strong absorption in the near−infrared region and lower photon energy loss during photocurrent generation^3,12–15^. With the PCE of PSCs approaching the commercially viable efficiencies, increased attention has been paid to the cost of the photovoltaic materials^16^. Recently, a variety of low-cost polymer donors, such as poly(thiophene-quinoxaline) derivatives^17^, have been well developed that can work well with SMAs, Therefore, the bottleneck for the commercialization of the PSCs, from the cost point of view, is the design and synthesis of low-cost SMAs.

The narrow bandgap SMAs possess a typical acceptor-donor-acceptor (A–D–A) or A–DA’D–A molecular structure with aromatic-fused-ring D or DA’D-core, like ITIC^18^ and Y6^19^. In the SMAs, the intramolecular charge transfer involving D–A interactions provide a powerful approach for tuning their absorption and energy levels, while the intermolecular interactions of the terminal A–units (the π–π stacking of the A–units) of the SMA offer the main electron channel in devices^5,20^. Regarding their synthesis, the SMAs are generally constructed with modular synthesis^21^, by the conventional pyridine-catalyzed Knoevenagel condensation of the aldehyde-terminated aromatic D or DA’D fused-ring^22–24^ with the activated methylene-based end-capping A-group^25–29^, such as 1,1-dicyanomethylene-3-indanone (IC) and its derivatives. However, the classical Knoevenagel condensation in the synthesis of the SMAs usually suffers from low yields (20–80%), long heating period (5–12 h) at 60 °C, unavoidable by-product due to using excess of A units (4-6 equiv. relative to D or DA’D unit) and thus tedious purifying work-up. Such drawbacks leads to high cost of the SMAs which limits their commercialization in PSCs.

To address the challenge, we demonstrated a Lewis acid-catalyzed atom-economic green synthesis, and found that the coupling of the aldehyde-terminated D unit and the A-end groups could be quantitatively finished the reaction within 15 min via a boron trifluoride etherate (BF_3_ ∙ OEt_2_) promoted Knoevenagel condensation in the presence of acetic anhydride. With this procedure, a gram-scale preparation of the widely used SMAs was demonstrated with an easier workup. Also, this method is convenient for the synthesis of new SMAs, especially those with new A-end groups. Most importantly, for the high-performance SMAs (such as Y6), their synthesis cost could be reduced by about 50% compared with conventional preparation. Thus our work provide a greener, large-scale and low-cost synthesis of the SMAs with non-metal catalyst at room temperature, which are desirable for commercialization of PSCs.

## Results

### Design with Knoevenagel condensation

The application of D–A concept to construct tailor-made organic semiconductors has greatly promoted the progress of organic electronics^30^, and various chemical strategies have been developed for their synthesis (Supplementary Fig. 1). Especially, Knoevenagel condensation holds a great potential for a greener and effective synthesis without toxic chemicals (such as tin compound) and without noble metal catalyst that usually used in the stille couple reaction. Herein, we mainly focus on improving Knoevenagel condensation for a facile synthesis of D–A or A–DA’D–A structured SMAs used in PSCs.

As an important classical reaction, Knoevenagel condensation, the coupling of carbonyl compounds with the active methylene groups typically carries out in the presence of a weak base as catalyst, such as pyridine^31^. And a variety of well-developed methodologies have been developed to promote such transformation, like using ionic liquid, Lewis acid or microwave irradiation. So far, the well-developed Knoevenagel condensation are mainly used in the synthesis of non-conjugated products, such as chemical intermediates, pharmaceuticals, cosmetics and perfumes^31^. In the field of organic semiconductors, such as the SMAs in PSCs discussed here, the carbonyl compounds and active methylene groups for the Knoevenagel condensation are extensively conjugated to tune their photovoltaic properties, thus inevitably causing steric hindrance of the aryl group and solubility issues in common polar solvents. In addition, due to the reversible nature of this condensation, large excess of the IC-based methylene groups (4-6 equiv. relative to D or DA’D unit) is usually required to push the reaction equilibrium in favor of the desired products. In this case, the IC-based methylene groups could self-condense and/or further react with the target Knoevenagel product^31,32^. Therefore, it is highly desirable to search for a more effective Knoevenagel condensation method for the synthesis of the organic semiconductors.

Given the high reaction activity of diacetate and its easier access from corresponding aldehyde^33–35^, we proposed the idea of conducting the Knoevenagel reaction via the diacetate toward a convenient synthesis of organic semiconductors. It is noted that Sakai et al. pioneeringly conceived a one-pot procedure to afford Knoevenagel products with the assistance of acetic anhydride reactant and catalyst. This procedure is successful in providing a good yield of ca. 70% for most of the Knoevenagel products after column chromatography^36^, suggesting an alternative route to promote the conventional Knoevenagel reaction. Despite the advantage, the promotion of the Knoevenagel reaction via a quantitative transformation under a short reaction time remains a major challenge in chemistry. In addition, metal-free protocol is more desirable for the synthesis of organic photovoltaic materials. Recently, it has been reported that the metal residue in the photovoltaic materials usually result in a poor device performance in PSCs^37,38^. To remove this concern, tedious purification by recrystallization, time-consuming column chromatography or multiple precipitation would inevitably increase the cost of the SMAs. After screening a variety of Lewis acid based catalysts, acid anhydrides and solvents in the Knoevenagel condensation, we found that BF_3_ ∙ OEt_2_ catalyst is remarkably highly effective as a non-metal catalyst in the synthesis of the SMAs via a quantitative and fast condensation of aromatic aldehyde with A-end groups even at room temperature.

### Reaction optimizations

**Fig. 1:**
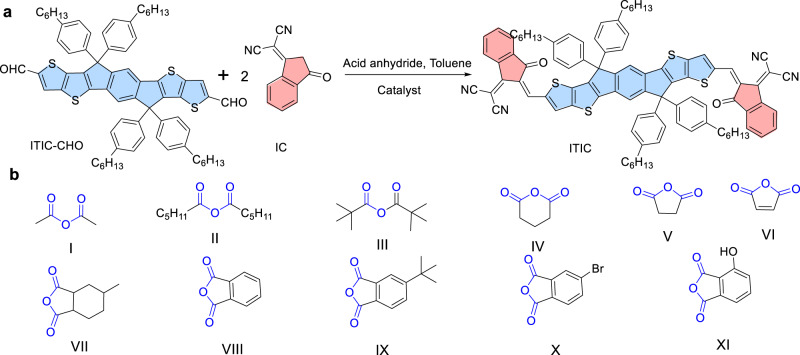
The synthesis of SMAs. **a** The synthetic rout of ITIC, the bule color represents D unit and the red color represents the A unit. **b** The acid anhydrides explored, acid anhydride groups are indicated with blue color.

As a landmark SMA widely used in the PSCs, ITIC was selected as the model compound to conduct our investigation (Fig. 1 and Table 1)^18^. Although the conventional Knoevenagel condensation led to ITIC with a low yield of 21% in the initial report, the concept of constructing such A–D–A structured SMAs opened an avenue for designing state-of-the-art SMAs. In our case, Knoevenagel condensation was initially conducted with the corresponding aldehyde (ITIC–CHO) and two equivalences of IC end group in toluene in presence of acetic anhydride at 60 °C. Obviously, no reaction took place without any catalyst. Then, a variety of Lewis acid catalysts were examined, including LiOTf, SnCl_2_, RuCl_3_, AlCl_3_, Yb(OTf)_3_, FeCl_3_, InCl_3_ and GaCl_3_. Among the Lewis acid catalysts, LiOTf, SnCl_2_ and RuCl_3_ were found to be ineffective for this condensation, most likely due to their poor capability in activating the aldehyde to form corresponding diacetate intermediate (entries 1-3, in Table 1). For AlCl_3_, Yb(OTf)_3_ and FeCl_3_, they showed poor catalytic performance, possibly due to their poor solubility in toluene (entries 4-6).

**Table 1:** The screening of the reaction conditions with metal catalysts for the synthesis of ITIC with 2 equiv. IC group^a^.

Entry	Catalyst	Catalyst (mol %)	*T* (°C)	Time (min)	Yield^b^ (%)
1	LiOTf	10	60	60	0
2	SnCl_2_	10	60	60	0
3	RuCl_3_	10	60	60	0
4	AlCl_3_	10	60	60	0
5	Yb(OTf)_3_	10	60	60	0
6	FeCl_3_	10	60	60	0
7	InCl_3_	10	0	60	10
8	InCl_3_	10	30	60	64
9	InCl_3_	1	60	300	20
10	InCl_3_	2	60	300	32
11	InCl_3_	5	60	125	>99%
12	InCl_3_	8	60	80	>99%
13	InCl_3_	10	60	40	>99%
14	InCl_3_	15	60	30	>99%
15	InCl_3_	20	60	30	>99%
16	GaCl_3_	10	0	60	18
17	GaCl_3_	10	30	88	80
18	GaCl_3_	1	60	150	>99%
19	GaCl_3_	2	60	80	>99%
20	GaCl_3_	5	60	30	>99%
21	GaCl_3_	8	60	13	>99%
22	GaCl_3_	10	60	10	>99%
23	GaCl_3_	15	60	9	>99%

^a^Reactions were performed with using ITIC-CHO and IC with a molar ratio of 1: 2;

^b^The yields were determined by ^1^H NMR spectroscopy (400 MHz) with toluene-d_8_ as an internal standard and solution.

Notably, InCl_3_ displayed good catalytic activity, while GaCl_3_ proved to be a more effective catalyst as the reaction could complete within a shorter time or at a lower loading amount (entries 11–23 in Table 1). As shown in Table 1, the reaction rate using GaCl_3_ as catalyst was 4 times faster than that using InCl_3_ as catalyst. With higher loading amount of catalyst, the reaction could go to completion in a shorter period. From Supplementary Fig. 2, it can be seen that there is a trade-off between reaction time and the concentration of the catalyst. Although reaction time can be shortened with a higher concentration of catalyst, which raised the concerns on the catalyst residuals. As a result, 5 mol % GaCl_3_ was selected as the optimal loading amount, which led to full reaction conversion within 30 min at 60 °C (entry 20 in Table 1). The different catalytic activities between GaCl_3_ and InCl_3_ may be related to their molecular nature^39,40^. As shown in Supplementary Fig. 3, GaCl_3_ adopts a bitetrahedral structure with two bridging chlorides. While for InCl_3_, it features octahedrally coordinate In (III) center with close-packed chloride arrangement. As a consequence, GaCl_3_ has a lower lattice energy, thus a better solubility (virtually all solvents) relative to that of InCl_3_. The solubility is one plausible reason for the high catalytic activities of GaCl_3_.

Despite the remarkable catalytic activity of GaCl_3_, metal-free protocol is more desirable as metal residue is usually associated with the poor device performance as mentioned above. Encouragingly, we found that just with simple and cheap BF_3_ ∙ OEt_2_ as catalyst, higher catalytic activity was demonstrated (Table 2), even at room temperature (entries 2–6 in Table 2). Thus our approach provides a non-metal-catalyzed Knoevenagel condensation and an efficient method towards the synthesis of SMAs. Interestingly, we found that even when the reaction was performed at a low temperature of 0 °C, the reaction could also be completed within 80 min, suggesting a low-energy-consuming approach (entry 1).

**Table 2:** The screening of reaction conditions with organic catalyst for the synthesis of ITIC with 2 equiv. IC group in toluene^a^.

Entry	Catalyst	Catalyst (equiv^b^)	*T* (°C)	Time (min)	Yield^c^ (%)
1	BF_3_ ∙ OEt_2_	5	0	60	>99%
2	BF_3_ ∙ OEt_2_	5	28	15	>99%
3	BF_3_ ∙ OEt_2_	1	28	30	>99%
4	BF_3_ ∙ OEt_2_	2	28	20	>99%
5	BF_3_ ∙ OEt_2_	8	28	10	>99%
6	BF_3_ ∙ OEt_2_	10	28	5	>99%
7	BF_3_ ∙ OEt_2_	1	60	10	>99%
8	BF_3_ ∙ OEt_2_	2	60	8	>99%
9	BF_3_ ∙ OEt_2_	5	60	5	>99%
10	BF_3_ ∙ OEt_2_	8	60	5	>99%
11	BF_3_ ∙ OEt_2_	10	60	5	>99%
12	BF_3_ ∙ THF	5	28	15	>99%
13	BF_3_ ∙ HOAc	5	28	15	>99%
14	BF_3_ ∙ CH_3_CN	5	28	15	>99%
15	Cyanuric chloride	10	60	60	0
16	Carbon bromide	10	60	60	0
17^*d*^	Pyridine	15	60	720	22
18^*e*^	Pyridine	15	60	360	83

^a^Reactions were performed using ITIC-CHO and IC with a molar ratio of 1:2.

^b^Relative to IC group.

^c^The yields were determined by ^1^H NMR spectroscopy (400 MHz) with toluene-d_8_ as an internal standard and solution.

^d^CDCl_3_ used as solvent, and 2 equiv. IC group was used.

^e^CDCl_3 _used as solvent, and 6 equiv. IC group was used.

Furthermore, different solvents, such as xylene, cyclohexane, pyridine, THF and CH_2_Cl_2_, were also screened for this GaCl_3_ and BF_3_ ∙ OEt_2_-catalyzed transformation (Supplementary Table 1). It was found that xylene and CH_2_Cl_2_ were suitable for this reaction. However, cyclohexane, pyridine and THF led to no desired product. The low solubility of those conjugated substrates in cyclohexane should be the main reason for its ineffectiveness, while the weak coordination effect of GaCl_3_ and BF_3_ ∙ OEt_2_ with the aldehyde substrate in pyridine and THF could explain why THF solvents were not applicable for this condensation. Besides BF_3_ ∙ OEt_2_, boron trifluoride tetrahydrofuran (BF_3_ ∙ THF), boron trifluoride-acetic acid (BF_3_ ∙ HOAc) and boron trifluoride acetonitrile (BF_3_ ∙ CH_3_CN) were also tested, they are as effective as that of BF_3_ ∙ OEt_2_ (entries 12–14 in Table 2). While for cyanuric chloride and carbon bromide (entries 15–16 in Table 2), they were found to be ineffective for this condensation, probably due to their poor capability in activating the aldehyde to form the diacetate intermediate. To better understand the high reaction activity of our method, we performed in situ NMR experiment for the conventional pyridine-catalyzed Knoevenagel condensation (Supplementary Fig. 5). With the adding of 2 eq IC and 1eq ITIC-CHO in the presence of pyridine, it can be seen that a lower conversion of 22% for ITIC was obtained after 12 h (entry 17 in Table 2). The result suggests that large excess of IC group is needed to push the equilibrium in favor of the final product in the conventional pyridine-catalyzed Knoevenagel condensation, confirming the high reaction activity of our BF_3_ ∙ OEt_2_-catalyzed method.

Then the effect of different anhydrides (Fig. 1b) on the reaction was examined (Fig. 1b, Supplementary Tables 2 and 3). As for the aliphatic carboxylic anhydrides (I-III), all reactions worked well for GaCl_3_ and BF_3_ ∙ OEt_2_, albeit the anhydrides with longer alkyl chain (II) and steric alkyl chain (III) required slightly extended reaction time. Regarding the cyclic anhydrides (X, VIII and IV), only those with low ring strain can lead to the full completion of the reaction for GaCl_3_ (Supplementary Table 2). While all of them are not effective for BF_3_ ∙ OEt_2_ (Supplementary Table 3). Our results indicate that aliphatic carboxylic anhydrides (I-III) are all potential candidates for the BF_3_ ∙ OEt_2_-catalyzed Knoevenagel condensation.

### Mechanistic studies

**Fig. 2:**
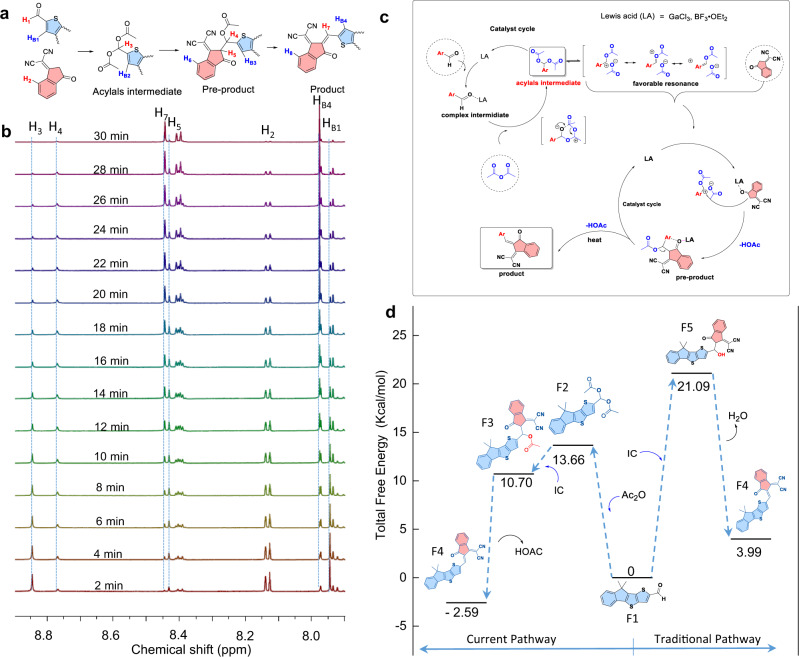
Mechanism studies. **a** The reaction subsequently shows starting materials (ITIC-CHO, IC), the diacetate intermediate and pre-product, as well as the ITIC product. **b** In situ ^1^H NMR (600 MHz) experiments in toluene-d_8_ with an expanded view in the fields of 9.5-7.8 ppm. During the experiment, a mixture of ITIC-CHO (5.0 mg) and IC (1.8 mg) in a molar ratio of 1: 2 in toluene-d_8_ (1 ml) was added into the NMR tube along with GaCl_3_ (about 1 mg) and acetic anhydride (0.02 ml). **c** Plausible reaction pathways for the Lewis acid-catalyzed Knoevenagel condensation. **d** Computed free-energy profiles for comparison of the Lewis acid-catalyzed pathway and the traditional pathway. The calculation was conducted with B3LYP-6-31G (d, p). Relative Gibbs free energies of the reaction system at 298 K are given in kcal/mol, whereas the energy of the starting material is arbitrarily set to zero. In the molecules, the bule color represents D unit and the red color represents the A unit.

The in situ ^1^H NMR (Fig. 2) show the gradual consumption of starting materials (ITIC-CHO, IC), the presence of the diacetate intermediate and pre-product, as well as the formation of the product (ITIC). Initially, the disappearance of the proton at 9.40 ppm (the aldehyde of ITIC-CHO) and the appearance of new proton at 8.84 ppm (H_3_ in Fig. 2a) confirmed the complete conversion of ITIC-CHO to its corresponding diacetate. As the reaction progress, the intensity of H3 from the gem-diacetate decrease, while new germinal protons (assigned to the pre-product) at 8.77 ppm (H4) and 8.42 ppm (H5) show up with the same intensity. The final product was observed on the basis of the new olefinic proton at 8.44 ppm (H7), and its intensity gradually increased with the disappearance of the typical protons for the intermediate and the pre-product. When the reaction turned to completion, the characteristic protons of the final product became the dominant peaks, suggestive of quantitative conversion of the starting materials to ITIC. The conclusion can also be supported by the evolution of the protons attached at the *β*-positions of the thiophene groups (H_B2_ from the diacetate at 7.97 ppm, H_B3_ from the pre-product at 7.94 ppm and H_B4_ from the product at 7.98 ppm), as a result of steric repulsion of the bulky groups attacked at the *α*-positions during the subsequent chemical transform.

Based on the above experiments and previous reports, the mechanism for the Lewis acid catalyzed one-pot reaction is illustrated in Fig. 2. Initially, the coordination of the carbonyl oxygen in ITIC-CHO with the Lewis acid generated a highly electrophilic carbonyl group, which was the primary driving force for such reaction. At this moment, significantly red shift was observed for the absorption of ITIC-CHO in toluene solution, suggestive of such coordination effect (Supplementary Fig. 4). Interestingly, the addition of THF or pyridine could lead to the recovery of its absorption, indicating the coordination of carbonyl group with Lewis acid was destroyed. This phenomenon could be used to explain the ineffectiveness of such condensation in THF or pyridine. Upon the coordination, the carbonyl group was attacked by the oxygen atom of acetic anhydride, and the subsequent intramolecular rearrangement gave the diacetate intermediate. Then, nucleophilic attack of the diacetate intermediate by the enolizable activated IC group led to the pre-product, which underwent elimination of acetic acid to afford the desired product along with release of the Lewis acid catalyst.

To gain more insights into the reaction, DFT calculations at the B3LYP/6-31G(d) level with the Gaussian 03 program package were carried out and the results are shown in Fig. 2d^41,42^. Notably, the reaction was simplified only with the consideration of the mono-aldehyde transformation for clarification, and thus F1 substrate was used as model compound for ITIC-CHO. In the Lewis acid-catalyzed pathway, it can be seen that converting aldehyde (F1) to the acylals intermediate (F2) is endothermic with a small energy barrier of 13.66 kcal mol^−1^, and the subsequent sequence from acylal intermediate (F2) to the final product (F4) is highly thermodynamically favorable, with the total Gibbs energy change of the process being about –2.59 kcal mol^−1^. However, in the conventional Knoevenagel condensation, the energy barrier to the alcohol intermediate (F5) was 21.09 kcal mol^−1^, which makes it harder to overcome. And it is a classical reversible process with an endothermic energy barrier by 3.99 kcal mol^−1^ from aldehyde (F1) to the final product (F4). This might explain that large excess of IC-based methylene groups was required to push the equilibrium in favor of the final products for the conventional Knoevenagel condensation in the viewpoint of thermodynamics.

### Scope of the method

**Fig. 3:**
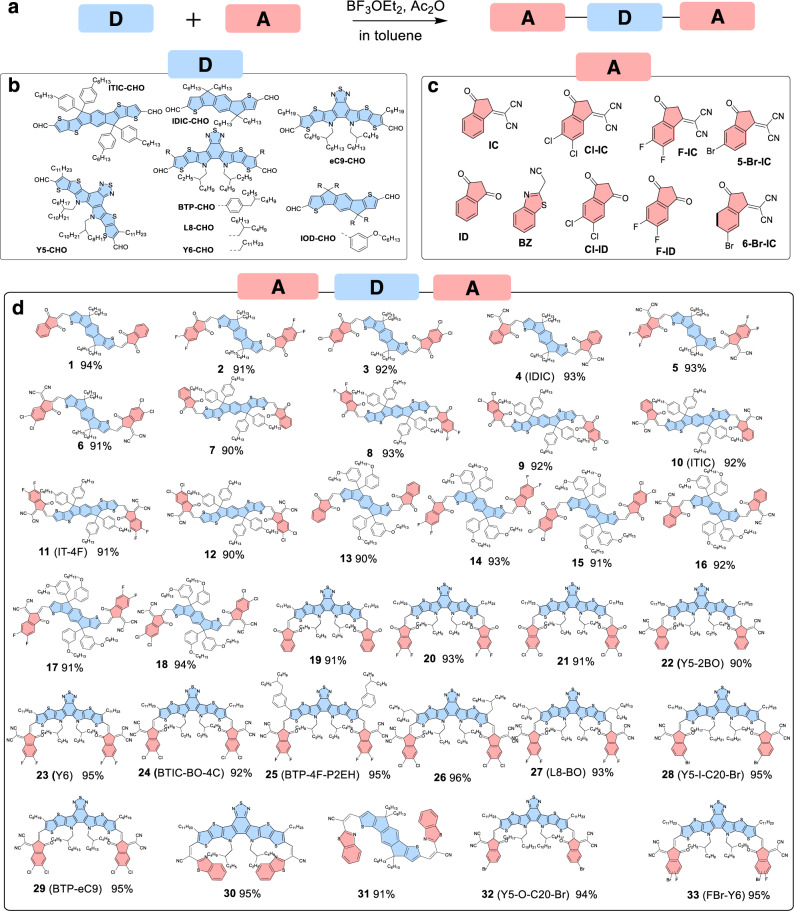
Chemical structures and synthetic routes. **a** The synthetic route for SMAs; **b** The aldehyde-terminated D groups; **c** The end-capping A groups; **d** Chemical structures of SMAs generated with isolated yields. To simplify, here the D-units represent aromatic-fused-ring D or DA’D-core. In the molecules, the bule color represents D unit and the red color represents the A unit. For the reported SMAs, the abbreviations of their name are given in the brackets.

With the newly established method in hand, we investigated the Knoevenagel condensation of various derivatives of ITIC-CHO with different side chains (and/or different aromatic-fused-rings) and the A units (Fig. 3). In general, the synthesis of all the SMAs were conducted in toluene at room temperature for 15 min with BF_3_ ∙ OEt_2_ as catalyst. When the alkoxyl group (like IDD-CHO) incorporated in the side chains or thiadiazole groups in the aromatic-fused-rings (like Y6-CHO), all reactions proceeded smoothly to give the desired products. Besides those reported SMAs, the method is convenient for the construction of new SMAs, especially those with new A-end groups (Fig. 3c). As for the conventional pyridine-catalyzed Knoevenagel condensation, the acidity of the methylene group is also an important factor related to the yields of the reaction. Generally, the IC-based A groups (IC, Cl-IC and F-IC) have a higher degree of acidity than that of dione-base A groups (ID, Cl-ID, and F-ID), and the halide substitution on A groups can increase their degree of acidity. Interestingly, there is no obvious difference on the reactions when different A-units were used in the BF_3_ ∙ OEt_2_-catalyzed reactions (Fig. 3c). The result suggests that the BF_3_ ∙ OEt_2_-catalyzed Knoevenagel condensation is an effective approach for the synthesis of the SMAs.

Comparing with the classical pyridine-catalyzed Knoevenagel condensation, in which large excess of IC or its derivatives was usually used to push the equilibrium in favor of the formation of the SMAs, our new method of the BF_3_ ∙ OEt_2_-catalyzed Knoevenagel condensation could quantitatively convert both starting materials to the corresponding products, providing an efficient and desirable approach for a low-cost synthesis of the SMAs, especially for those high-performance fluorinated ones, such as Y6 (23, in Fig. 3d). Recent studies revealed that fluorinated SMAs could suppress nonradiative triplet loss to achieve a better device performance^43,44^. However, their conventional synthesis required the use of excessive costly fluorinated IC end groups (such as F-IC), which significantly impeded their real application. In addition, fluorinated IC (such as F-IC) exhibits higher reaction reactivity than its non-fluorinated counterpart (such as IC) due to the higher degree of acidity of the methylene group. Thus they could further react with the target Knoevenagel SMA product, as evidenced by the synthesis of IT-4F using conventional pyridine-catalyzed Knoevenagel condensation (Supplementary Fig. 6). While using our BF_3_ ∙ OEt_2_-catalyzed Knoevenagel condensation, such phenomenon was not observed.

As a matter of fact, our new method of the BF_3_ ∙ OEt_2_-catalyzed Knoevenagel condensation could offer the pure final products through a simple reaction work-up, i.e., precipitation of the reaction mixture in methanol or ethanol followed by wash. Such a fast and quantitative transformation, together with its facile work-up, provide a convenient and economical procedure for a large-scale and low-cost preparation of the SMAs, which could potentially pave the way for their commercialization. As a typical example, a video of the synthesis of IT-4F (10, in Fig. 3d) is provided in Supplementary Video 1. To demonstrate this application, a gram-scale synthesis of Y6 was conducted with a high yield of 98%, while the initial reported yield of Y6 was only 64% at a miligram-scale^19^ using classical pyridine-catalyzed Knoevenagel condensation. And the reaction is convenient for further scale-up if needed. With the model for cost calculation^45^, the cost of material (C_g_, cost-per-gram) for ITIC, IT-4F and Y6 obtained with our method are 164.8 $ g ^−1^, and 192.3 $ g ^−1^ and 410.11 $ g ^−1^ (Supplementary Tables 5–7), all of which are thus reduced nearly by 50% relative to those using the conventional method. The result indicates that our modified strategy is helpful to provide low-cost photovoltaic acceptors for large-scale production.

### Photovoltaic properties

**Fig. 4:**
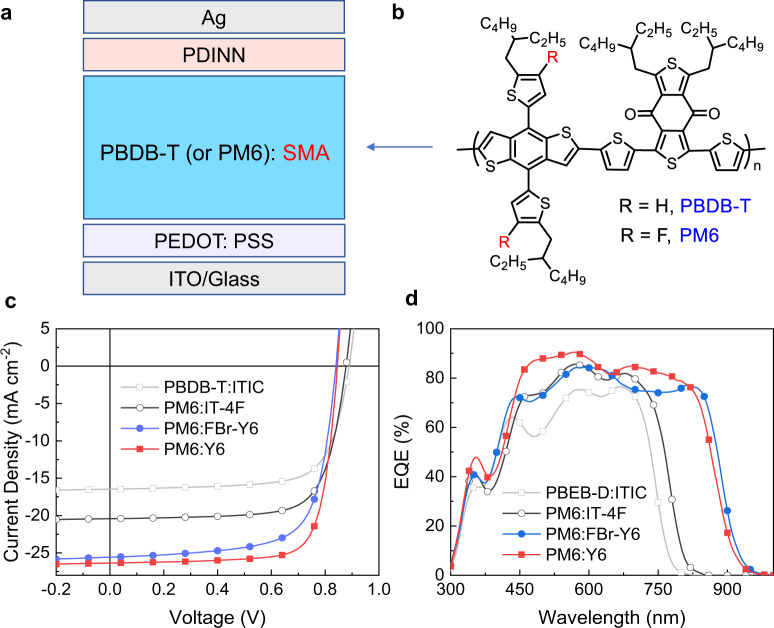
Photovoltaic performance of the PSCs. **a** Device structure of the PSCs. **b** Chemical structure of polymer donors; **c** *J–*V curves of the best PSCs based on PM6: SMAs (ITIC, IT-4F, FBr-Y6 and Y6, synthesized by BF_3_ ∙ OEt_2_-catalyzed Knoevenagel condensation) blend under the illumination of AM 1.5 G, 100 mWcm^−2^. **d** EQE spectra of the best devices.

For organic photovoltaic materials, besides their molecular structure, their purity is also an important factor determining the device performance. To improve the purity of SMAs, multiple column chromatography separations are needed in the conventional Knoevenagel condensation. This procedure is much more energy consuming, although they are effective to remove batch to batch variation in device performance. With the new method prepared SMAs (ITIC, IT-4F, FBr-Y6 and Y6) as acceptor, their photovoltaic performance was examined with PM6 or PBDB-T as donor (Fig. 4c). From Fig. 4 and Supplementary Table 8, it can be seen that all the SMAs show high PCE values, 10.66% for ITIC, 13.08% for IT-4F, 15.09% for FBr-Y6 and 17.15 % for Y6. The external quantum efficiency (EQE) spectra were measured to examine the spectral responses of the devices (Fig. 4d). The photo response range are related with optical bandgap of the SMAs, and the calculated short circuit current density (*J*_SC_) values for the IPCE curves agree well with the *J*_SC_ values obtained from the *J*–*V* curves. For the SMAs of ITIC, IT-4F and Y6, their PCE values are comparable to those of their corresponding state-of-the-art devices reported in literatures^18,19,46^. The results further confirm that our BF_3_ ∙ OEt_2_-catalyzed reaction with the simple work-up procedure could provide the final product with good quality for the high-performance PSCs.

## Discussion

A BF_3_ ∙ OEt_2_-catalyzed Knoevenagel condensation was developed for a fast and quantitative chemical synthesis of the A–D–A or A–DA’D–A structured SMAs, starting from aldehyde-terminated D or DA’D-units and end-capping A-units in the presence of acetic anhydride. Compared with the classical pyridine-catalyzed Knoevenagel condensation, this BF_3_ ∙ OEt_2_-catalyzed Knoevenagel condensation provides a thermodynamically favored path to the synthesis of the SMAs in quantitative yields through the germinal diacetate intermediate at a low temperature, and a variety of SMAs were synthesized with a convenient work-up. A gram-scale preparation was also demonstrated for Y6, which could be about 50% cost-saving compared with conventional preparation. Furthermore, our results further confirm that the simple work-up procedure could provide the final product with good quality for the high-performance PSCs. The advantages of fast and quantitative transformation together with an easier workup make the BF_3_ ∙ OEt_2_-catalyzed Knoevenagel condensation as a convenient and greener procedure for a large-scale and low-cost preparation of the SMAs, which are desirable for commercialization of PSCs. Beside the application in SMAs, this method can be also applied to the low-cost synthesis of other photovoltaic donors, such as those D–A structured small molecular donors^30^. Thus the widely application of such methodology will certainly accelerate the commercialization of PSCs. As the Knoevenagel condensation is among the most fundamental and strategically important transformation to construct D–A semiconductors, this methodology also offers a modular and robust synthesis of tailed-made D–A conjugated organic and polymeric materials for a wide range of emerging technologies, such as thin film transistors, light-emitting diodes, as well as memory devices, and near infrared image sensors or chemotherapy.

## Methods

### Reagents and materials

Reaction progress was monitored using a thin-layer chromatography (glass backed, extra hard layer, 60 Å, 250 μm thickness, F254 indicator). All chemicals were obtained from commercially available sources and used without further purification. Solvents are used as received. All procedures were carried out under open atmosphere with no precautions taken to exclude ambient moisture. IC, Cl-IC, FBr-IC and Y6 (used as reference) were purchased from Shenzhen Yirou Photovoltaic Technology Co., Ltd (eFlexPV), and PM6 (used in device), ITIC-CHO, IDIC-CHO, IOD-CHO were purchased from Solarmer Materials. Y6-CHO was purchased from hyper. Inc. F-IC, F-ID, Cl-ID were purchased from SunTech Inc. ID and BZ were purchased from Bide Pharmatech Ltd. BTP-CHO, eC9-CHO, L8-CHO and Y5-CHO were synthesized according to the method of reference^47^. 5-Br-IC and 6-Br-IC were synthesized according to the method of references^48,49^.

### General producer for the synthesis of SMAs

The aldehyde-terminated D units (0.10 mmol) and the activated methylene based IC units (0.20 mmol) were dissolved in toluene (5 mL). BF_3_ ∙ OEt_2_ (5 equiv of the IC units) and acetic anhydride (0.1 ml) were added, and the reaction mixture was stirred at room temperature for 15 min. Then, the reaction mixture was added dropwise into methanol with stirring. The precipitate was collected, washed with methanol several times, then dissolved in chloroform and added dropwise into methanol again to afford product. This process was repeated several times until the target SMAs was pure enough for photovoltaic devices. While for other SMAs (2F-Ic-IOD, 2Cl-Ic- IOD, Ic- IOD, 2F-YT- IOD, 2Cl-YT- IOD and YT- IOD, structure in Fig. 3), ethanol was used instead of methanol. For the application in organic electronics, it is suggested that sufficient stirring of the SMAs precipitation must be ensured. Also, it is a straightforward procedure, just by filtration of the SMA solution (in dichloromethane) though a short pad of silica gel to avoid time-consuming stirring.

### Gram-scale synthesis of Y6

The Y6-CHO (1.00 g, 0.95 mmol) and the F-IC (0.45 g, 0.47 mmol) were dissolved in toluene (30 mL). BF_3_ ∙ OEt_2_ (0.5 mL, 2.5 eq) and acetic anhydride (1.0 ml) were added, and the reaction mixture was stir at room temperature for 15 min. Then, the reaction mixture was dropwise into methanol, and the precipitate was collected as a crude product. The crude product was dissolved in chloroform and dropwise into methanol again to afford product (1.39 g, yield 98%).

### Characterizations

Proton (^1^H NMR) was recorded on Bruker AVQ-400 spectrometers using CDCl_3_ or toluene-d_8_ as solvents. While for the in situ experiment, ^1^H NMR spectra were measured on a JNM-ECZ600R/S3 (Jeol, Japan) (600 MHz). Chemical shifts (δ) are referred to internal Me_4_Si (0 ppm) as the standard, and are reported in ppm relative to the residual solvent signal (δ 7.26 for ^1^H NMR in CDCl_3_, δ 7.09, 7.00, 6.89 for ^1^H NMR in toluene-d_8_). Data for ^1^H was reported as follows: chemical shift (δ ppm), multiplicity (s = singlet, d = doublet, t = triplet, q = quartet, quint = quintet, hept = heptet, m = multiplet, br = broad), coupling constant (Hz), integration. The yields were determined by ^1^H NMR spectroscopy (400 MHz) in toluene-d_8_ solvent, with its methyl group used as an internal standard to calculate the quantity of the substrates. This calculation is based on the ratio of the integrated peaks of aldehyde proton of the substrates (or the olefinic proton for SMA product) and the methyl group in toluene-d_8_ solvent. The UV–vis absorption spectra were measured by Hitachi U-3010 UV–vis spectrophotometer. Malid-tof-ms: MALDI measurements were performed with MALDI-TOF MS Bruker Autoflex II.

### Device fabrication and characterization of the OSCs

The OSCs were fabricated with a structure of ITO/PEDOT: PSS/active layer/cathode interlayer (CIM)/Ag. The ITO glass was cleaned by sequential ultrasonic treatment in water, deionized water, acetone and isopropanol, and then treated in an ultraviolet ozone cleaner (Ultraviolet Ozone Cleaner, Jelight Company, USA) for 20 min. The PEDOT: PSS aqueous solution (Baytron P 4083 from H. C. Starck) was filtered through a 0.45 mm filter and then spin-coated on pre-cleaned ITO-coated glass at 4000 rpm for 30 s. Subsequently, the PEDOT: PSS film was annealed at 150 °C for 20 min in air to form a 30 nm film. A blend solution of PM6 or PBDB-T donor and SMA was prepared by dissolving the materials in chloroform, and then was spin-coated at 4000 rpm onto the PEDOT: PSS layer. Then methanol solution of CIM (PDINN) at a concentration of 1.0 mg mL^−1^ was deposited on the active layer at 3000 rpm for 30 s to afford a cathode buffer layer. Finally, the metal cathode Ag was thermal evaporated under a mask at a base pressure of approximately 10^−5^ Pa. The photovoltaic area of the device is 4.6 mm^2^. Optical microscope (Olympus BX51) was used to define the active area of the devices. The current density-voltage (*J*-*V*) characteristics of the OSCs were measured in a nitrogen glove box with a Keithley 2450 Source Measure unit. Oriel Sol3A Class AAA Solar Simulator (model, Newport 94023A) with a 450 W xenon lamp and an air mass (AM) 1.5 filter was used as the light source. The light intensity was calibrated to 100 mW cm^−2^ by a Newport Oriel 91150V reference cell. The voltage step and delay time were 10 mV and 1 ms, respectively. The scan started from −1.5 V to 1.5 V. The EQE was measured by Solar Cell Spectral Response Measurement System QE-R3-011 (Enli Technology Co., Ltd., Taiwan). The light intensity at each wavelength was calibrated with a standard single-crystal Si photovoltaic cell.

### Reporting summary

Further information on research design is available in the Nature Research Reporting Summary linked to this article.

## Supplementary information

Supplementary Information

Description of Additional Supplementary Files

Supplementary Video 1

Solar Cells Reporting Summary

## Data Availability

The data that support the findings of this study are available from the corresponding author on request.
