# Supplementary material for: Low-cost synthesis of small molecule acceptors makes polymer solar cells commercially viable
Source: Nat Commun. 2022 Jun 27;13:3687. doi: 10.1038/s41467-022-31389-y (PMC9237043; doi:10.1038/s41467-022-31389-y)
Supplement: Supplementary file 1 — Supplementary Information [file 41467_2022_31389_MOESM1_ESM.pdf]

Supplementary Information

Low-Cost Synthesis of Small Molecule Acceptors via a Quantitative and Fast Knoevenagel Condensation Makes Polymer Solar Cells Commercially Viable

Fu, *et al*.

Supplementary Figures

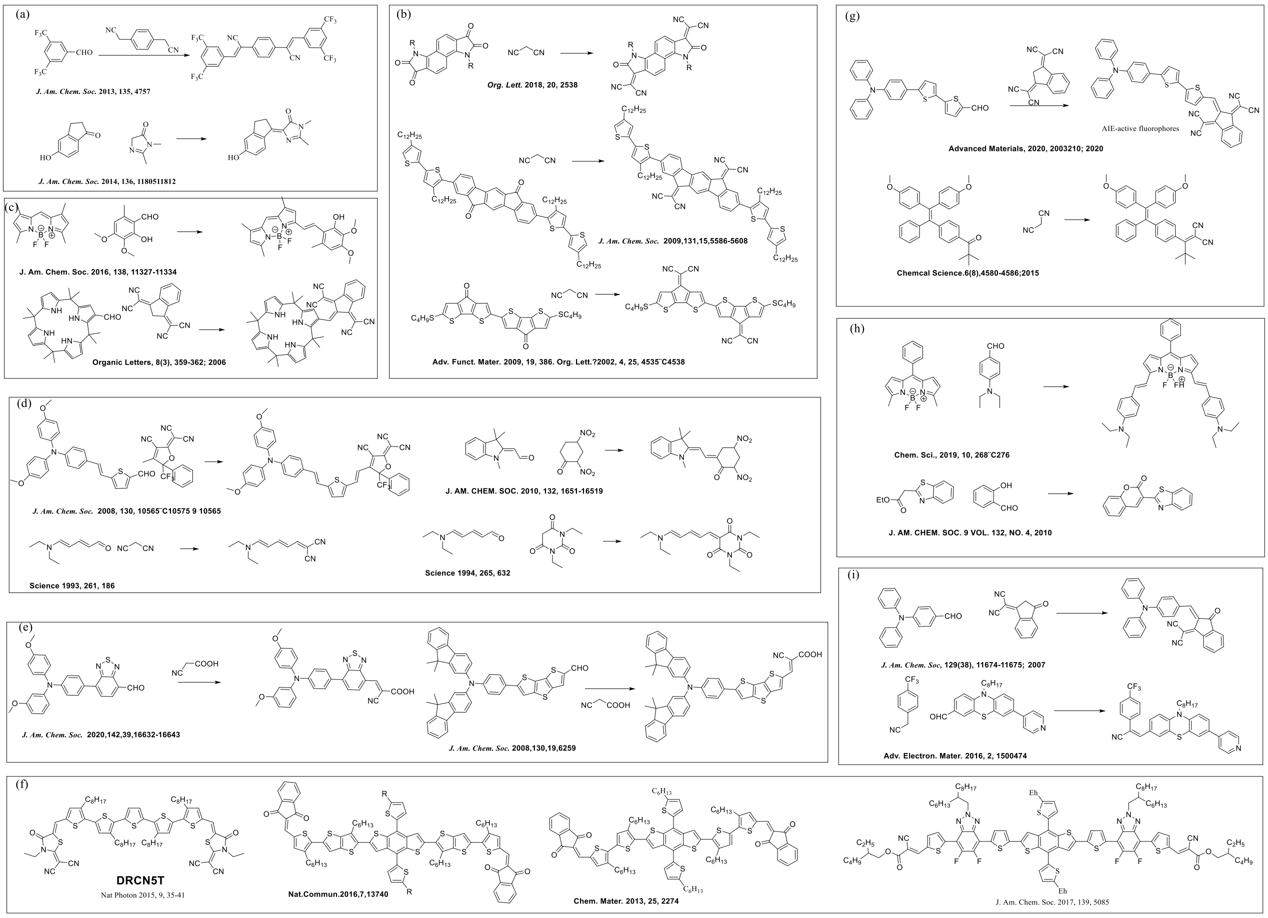

Supplementary Figure 1. Typical organic semiconductors constructed with the Knoevenagel condensation. (a) Organic light-emitting diodes; (b) Organic field-effect transistor. (c) Chemical senser; (d) Organic nonlinear optical; (d) Perovskite and dye-sensitized solar cells. (f) Photovoltaic donors; (g) AIE active fluorophore.(h) NIR image sensor; (i) Memory devices.

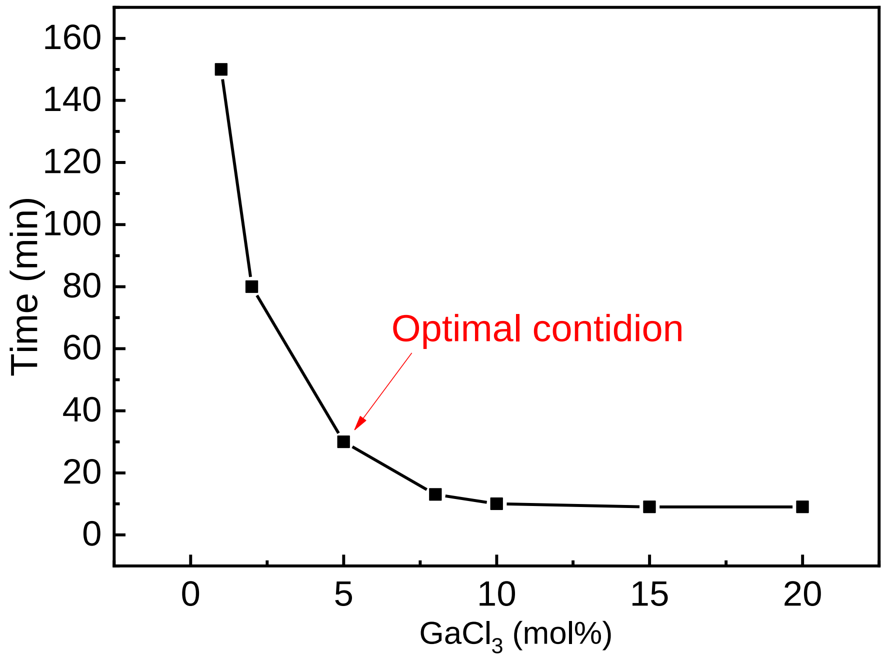

Supplementary Figure 2 The reaction time versus concentration of the catalyst, data was obtained from Table 1.

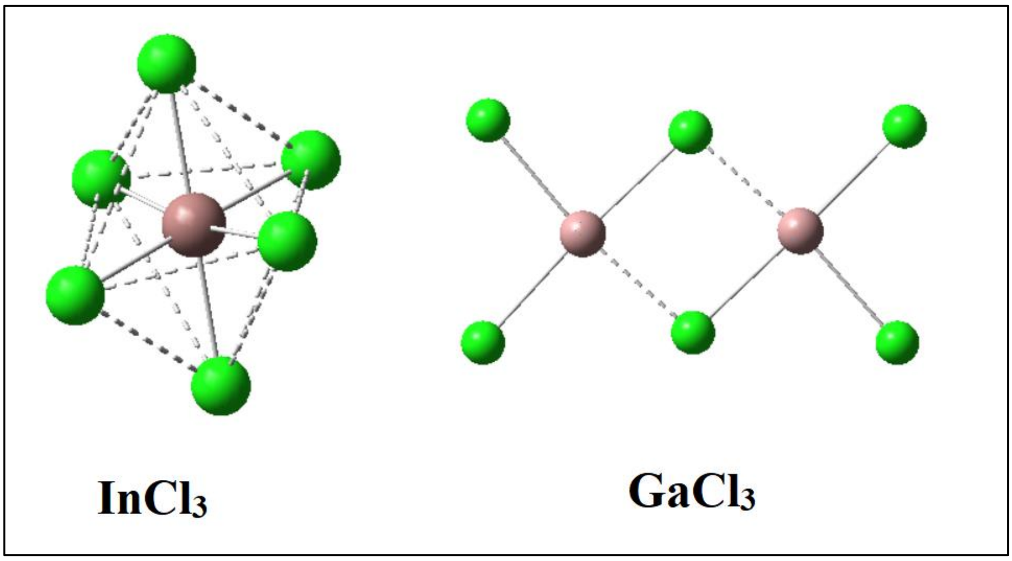

Supplementary Figure 3. Crystal structure of $InCl_3$ and $GaCl_3$. For $GaCl_3$, it is the chemical compound, and it adopts a bitetrahedral structure with two bridging chlorides. While $InCl_3$, It features octahedrally coordinate In (III) centers with close-packed chloride arrangement. As a consequence of its molecular nature and associated low lattice energy, $GaCl_3$ has a lower melting point vs $InCl_3$. $GaCl_3$ is soluble in virtually all solvents (such as toluene, CCl_4), even alkanes, which is truly unusual for a metal halide. While $InCl_3$ is only soluble in polar solvent, such as THF, ethanol.

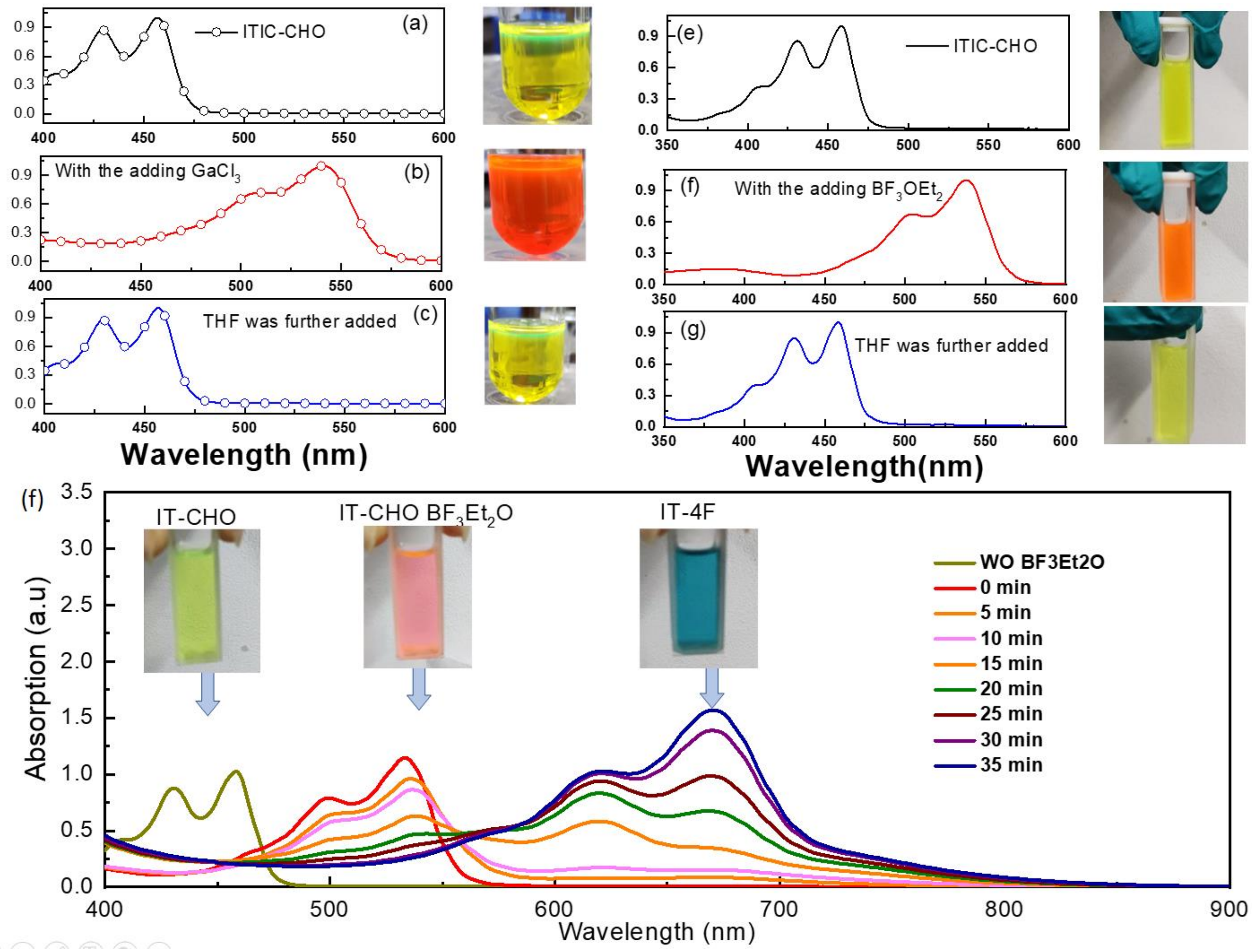

Supplementary Figure 4. (a, e) The absorption of ITIC-CHO in toluene. With the adding of (b) $GaCl_3$ or (f) $BF_3{\cdot}OEt_2$, the significantly red shifted absorption of ITIC-CHO in toluene solution is suggestive of coordination of the carbonyl oxygen in ITIC-CHO to $GaCl_3$ and $BF_3{\cdot}OEt_2$, (c, g) which can be easily destroyed by the addition of THF as can be clearly evidenced by the recovery of its absorption. This phenomenon can also explain the ineffective of such reaction in THF and pyridine, which may have a stronger interaction with the aldehyde that with $GaCl_3$. (f). the in-situ absorbance of the reaction mixture.

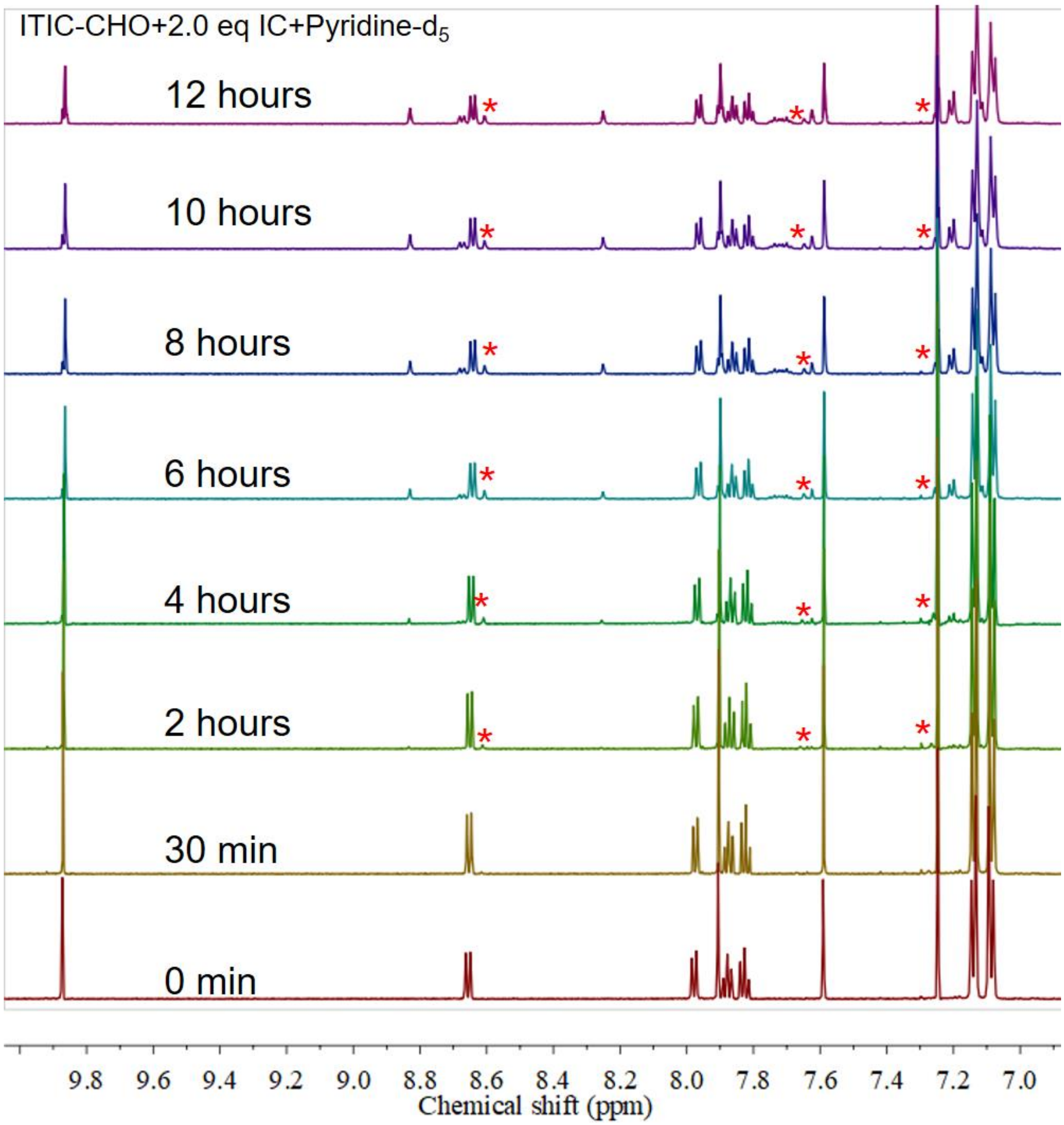

Supplementary Figure 5. In-situ NMR experiment for the conventional pyridine-catalyzed Knoevenagel condensation, with the adding of 2 eq IC and 1eq ITIC-CHO in the presence of pydine-d_5 as catalyst.

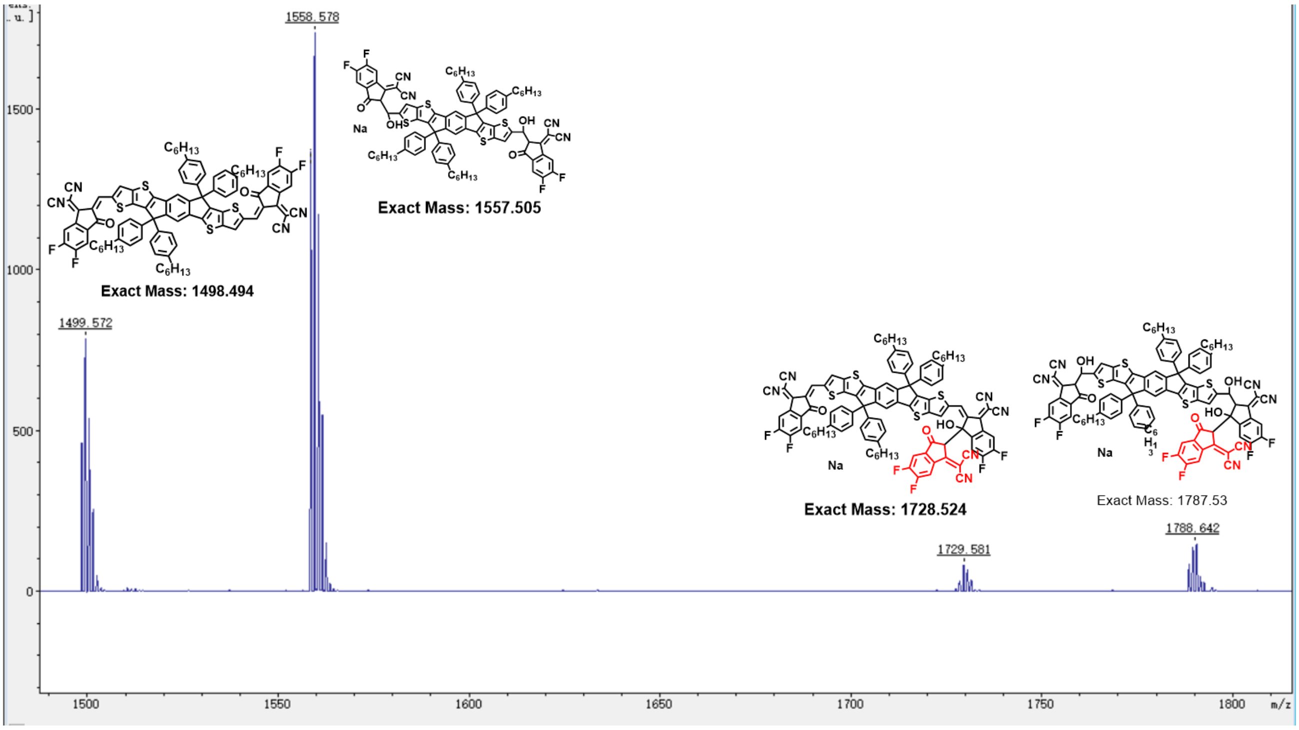

Supplementary Figure 6. MALDI-TOF MS of the reaction mixture of IT4F.

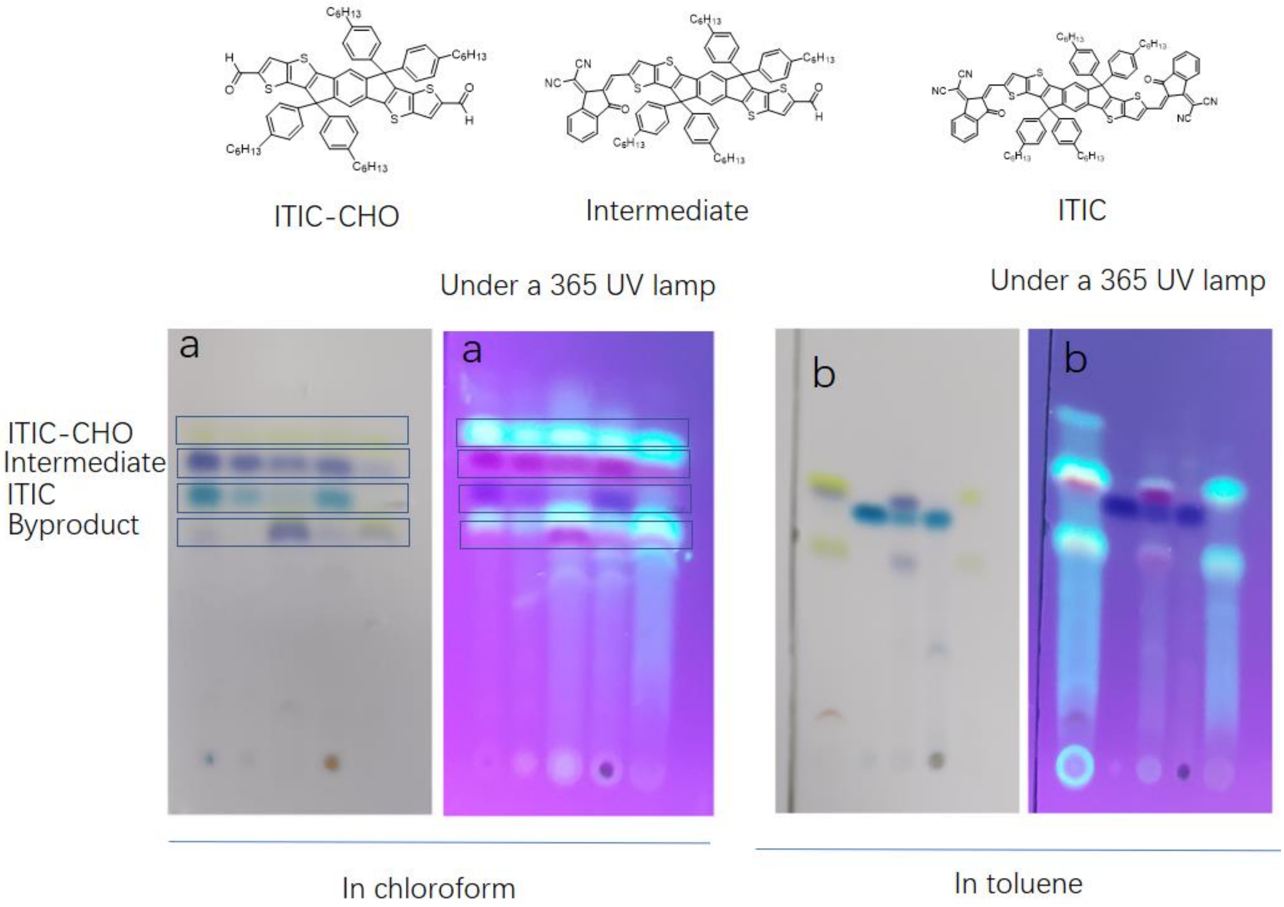

Supplementary Figure 7. TLC chromatograms for the visualization of the reactions. In each plate, the spots represent reactions with different catalysts from left to right: $AlCl_3$ (1), $GaCl_3$ (2), $FeCl_3$ (3), $InCl_3$ (4), $Yb(OTf)_3$ (5). When with chloroform as solvent, Plate A and Plate B are photographs before after irradiation under a 365 UV lamp; When with toluene as solvent, Plate C and Plate D are photographs before after irradiation under a 365 UV lamp. It can be seen that in Plate C the dark blue spot is the dominated spot.

NMR spectra of SMAs:

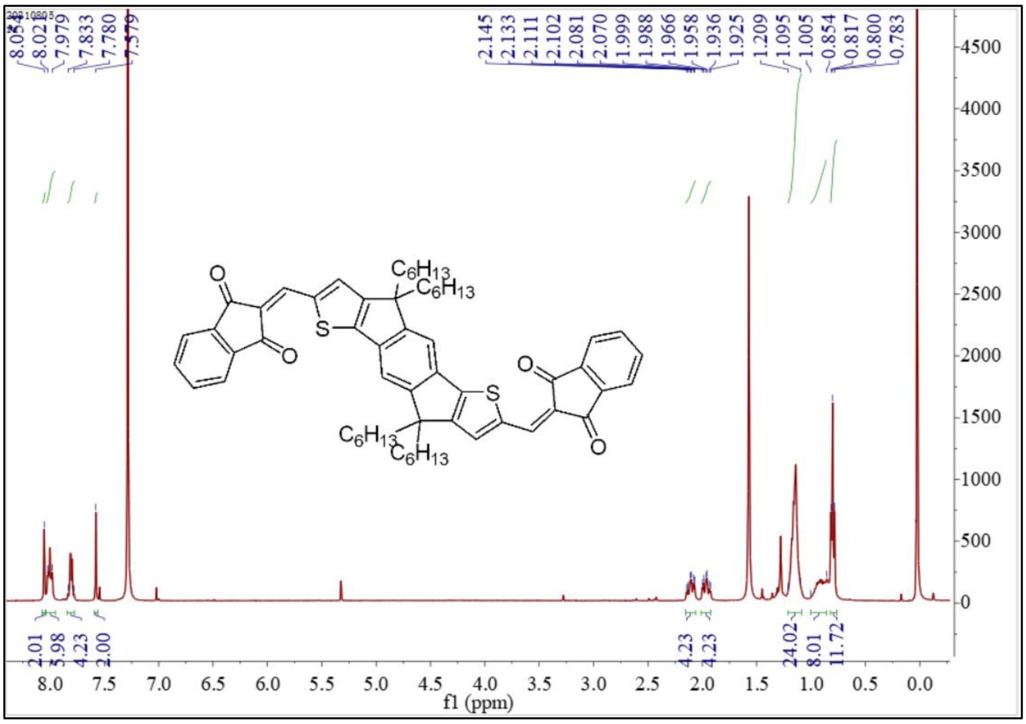

Supplementary Figure 8. ^{1}H NMR spectrum of **compound 1** . ^{1}H NMR (400 MHz, $CDCl_3$) δ 8.05 (s, 2H), 8.02-7.80 (m, 6H), 7.83-7.78 (m, 4H),7.58 (s, 2H), 7.62 (s, 2H), 2.10 (dt, J = 3.6 Hz, J = 13.6 Hz, 4H), 1.96 (dt, J = 3.6 Hz, J = 13.6 Hz, 4H).1.20-1.07 (m,24H), 0.96-0.85 (m,8H),0.81 (t, J = 7.2 Hz,12H). MS (MADIL-TOF) m/z: $[M]^+$: calcd for $C_{60}H_{66}O_4S_2$; found $[M+1]^+$:915.415.

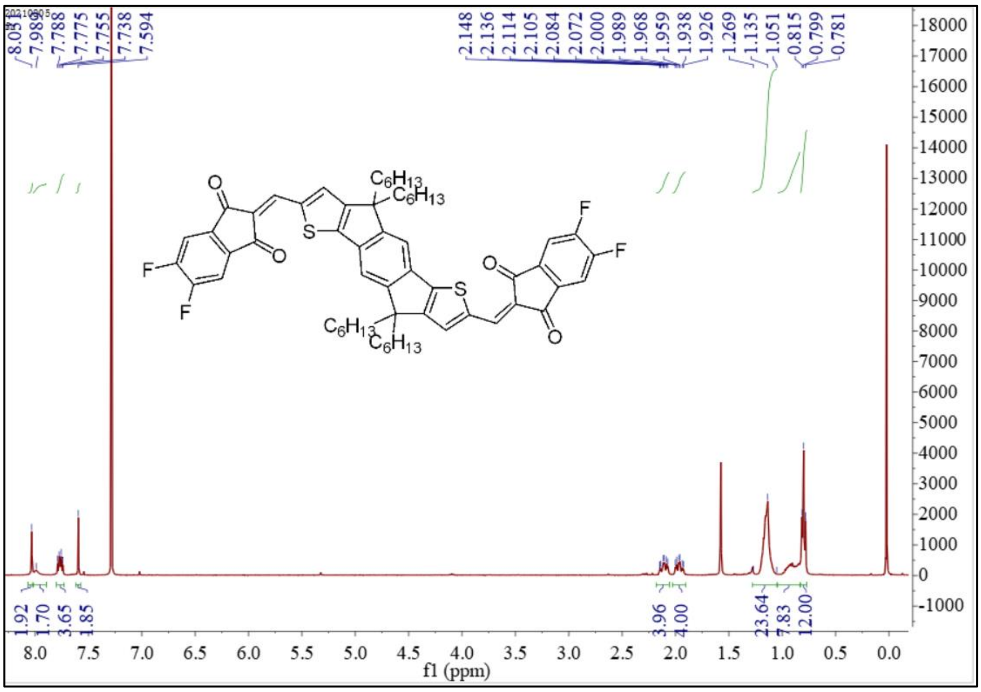

Supplementary Figure 9. ^{1}H NMR spectrum of **compound 2**. ^{1}H NMR (400 MHz, $CDCl_3$) δ 8.03 (s, 2H), 7.99 (s, 2H) 7.76-7.73 (m, 4H),7.75 (s, 2H), 7.62 (s, 2H), 2.10 (dt, J = 3.6 Hz, J = 13.6 Hz, 4H), 1.96 (dt, J = 3.6 Hz, J = 13.6 Hz, 4H).1.20-1.07 (m,24H), 0.96-0.85 (m,8H),0.81 (t, J = 7.2 Hz,12H). MS (MADIL-TOF) m/z: $[M]^+$: calcd for $C_{60}H_{62}F_4O_4S_2$; found $[M+1]^+$: 987.491.

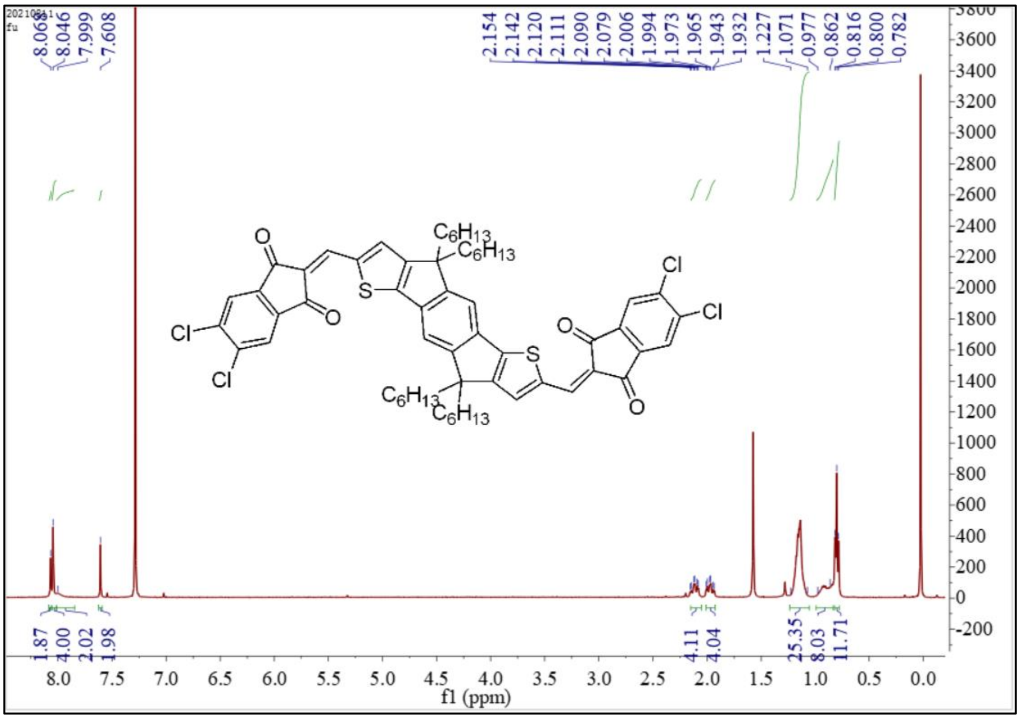

Supplementary Figure 10.^{1}H NMR spectrum of **compound 3**. ^{1}H NMR (400 MHz, $CDCl_3$) δ 8.07 (s, 2H), 8.05 (s, 4H), 8.00 (s, 2H),7.61 (s, 2H), 2.10 (dt, J = 3.6 Hz, J = 13.6 Hz, 4H), 1.96 (dt, J = 3.6 Hz, J = 13.6 Hz, 4H).1.20-1.07 (m,24H), 0.96-0.85 (m,8H),0.81 (t, J = 7.2 Hz,12H). MS (MADIL-TOF) m/z: $[M]^+$: calcd for $C_{60}H_{62}Cl_4O_4S_2$; found $[M+1]^+$:1051.371.

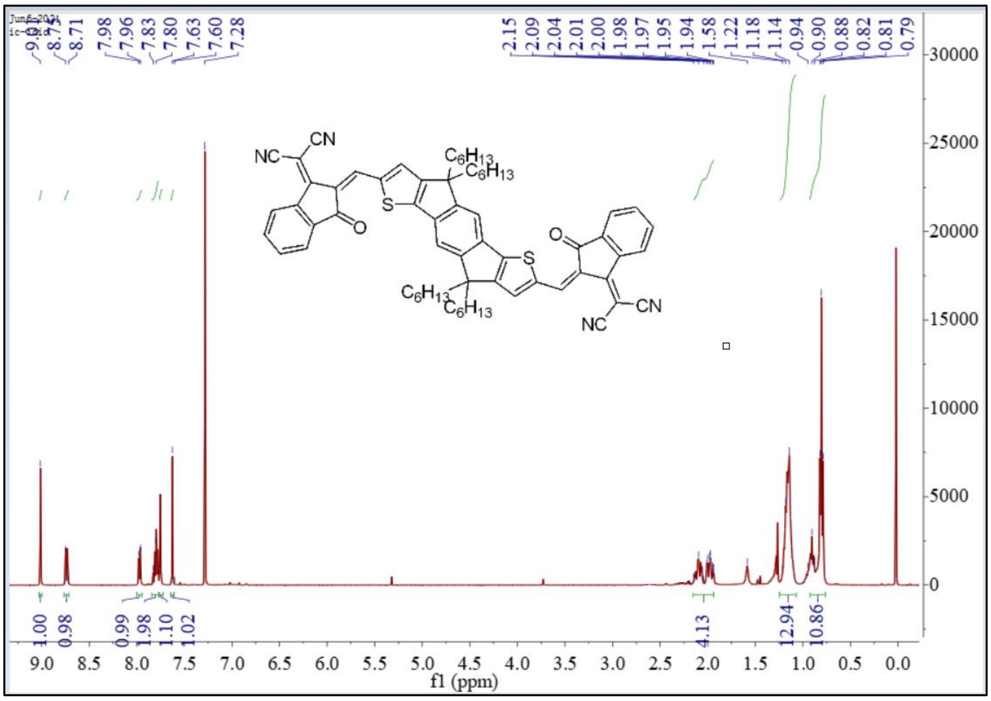

Supplementary Figure 11. ^{1}H NMR spectrum of **compound 4**. ^{1}H NMR (400 MHz, $CDCl_3$) δ 9.01 (s, 2H), 8.74 (dd, J = 1.2 Hz, J = 6.0 Hz 2H), 7.97 (s, dd, J = 0.8 Hz, J = 6.4 Hz 2H), 7.83-7.78 (m, 4H),7.75 (s, 2H), 7.62 (s, 2H), 2.10 (dt, J = 3.6 Hz, J = 13.6 Hz, 4H), 1.98 (dt, J = 3.6 Hz, J = 13.6 Hz, 4H).1.20-1.07 (m,24H), 0.96-0.85 (m,8H),0.81 (t, J = 7.2 Hz,12H). MS (MADIL-TOF) m/z: [M]+: calcd for $C_{66}H_{66}N4O_2S_2$; found $[M^{+1}]$: 1011.583.

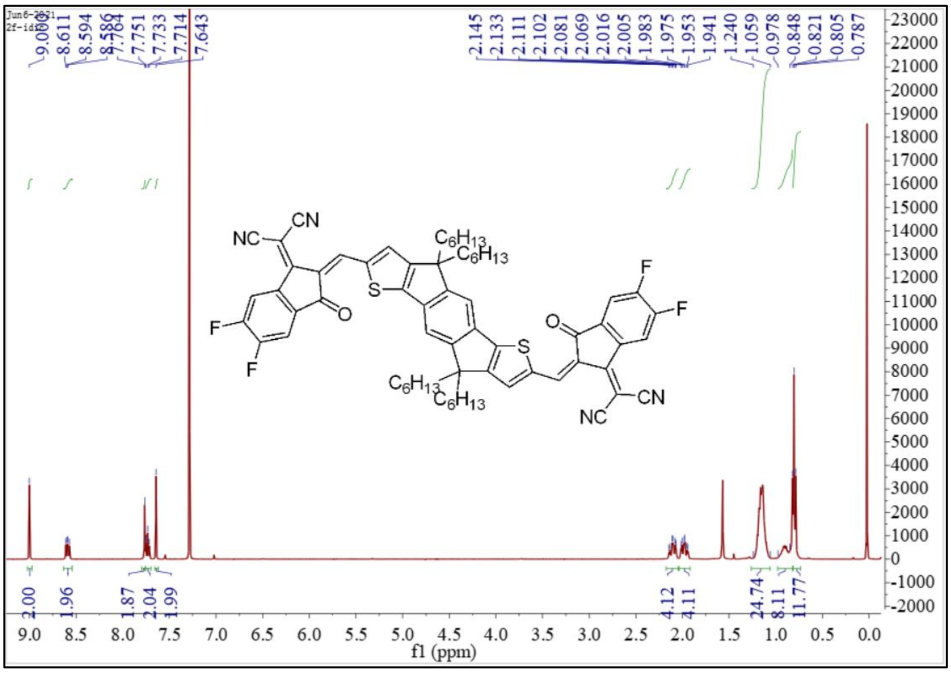

Supplementary Figure 12. ^{1}H NMR spectrum of **compound 5**.^{1}H NMR (400 MHz, $CDCl_3$) δ 9.00 (d, 2H), 7.45 (dd, J = 6.8 Hz, J = 10.0 Hz, 2H), 7.76 (s, 2H), 7.73 (t, J = 7.6 Hz 2H), 7.64 (s,2H), 2.06 (dt, J = 3.6 Hz, J = 13.6 Hz, 4H), 1.98 (dt, J = 3.6 Hz, J = 13.6 Hz, 4H).1.24-1.06 (m,24H), 0.98-0.85 (m,8H),0.81 (t, J = 7.2 Hz,12H). MS (MADIL-TOF) m/z: $[M]^+$: calcd for $C_{66}H_{62}F_4N_4O_2S_2$; found $[M+1]^+$: 1083.437.

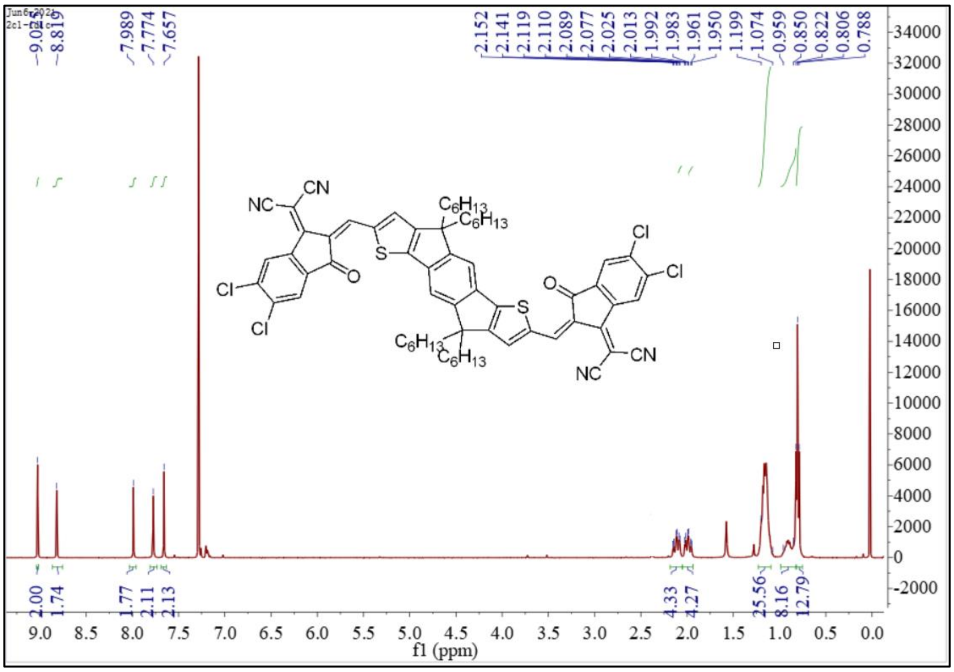

Supplementary Figure 13. ^{1}H NMR spectrum of **compound 6**. ^{1}H NMR (400 MHz, $CDCl_3$) δ 9.02 (s, 2H), 8.82 (s, 2H), 7.99 (s, 2H), 7.77 (s, 2H),7.66 (s, 2H), 2.11 (dt, J = 3.6 Hz, J = 13.6 Hz, 4H), 1.99 (dt, J = 3.6 Hz, J = 13.6 Hz, 4H).1.20-1.07 (m,24H), 0.96-0.85 (m,8H),0.81 (t, J = 7.2 Hz,12H). MS (MADIL-TOF) m/z: $[M]^+$: calcd for $C_{66}H_{62}Cl_4N_4O_2S_2$; found $[M+1]^+$: 1147.316.

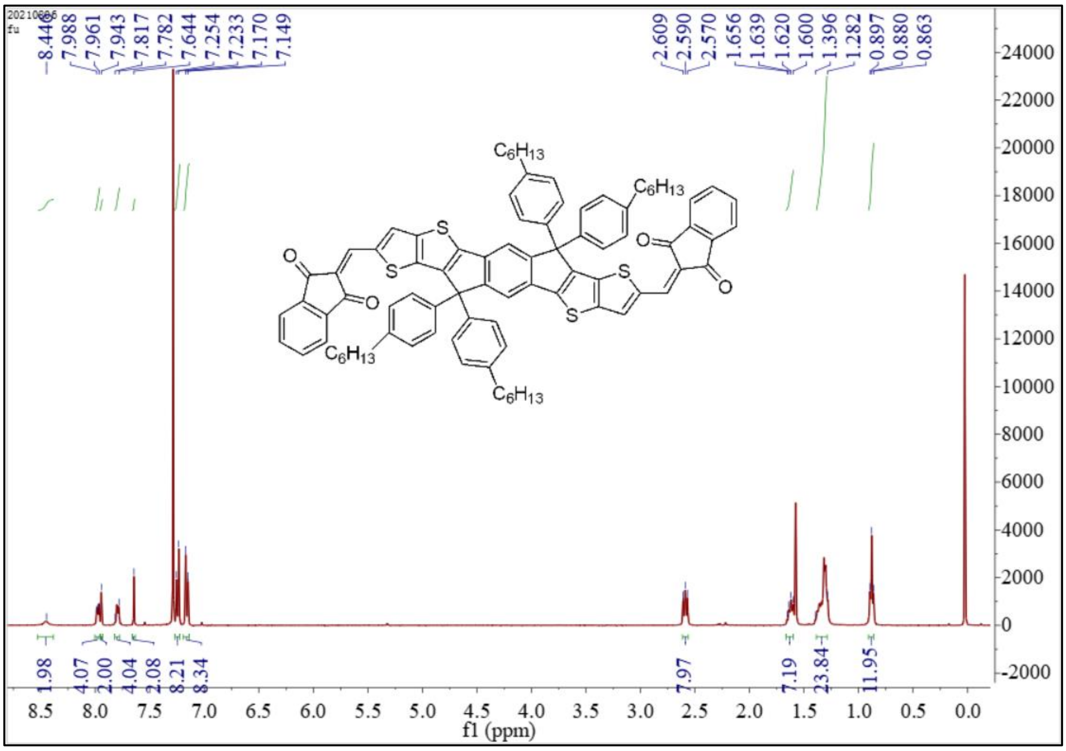

Supplementary Figure 14. ^{1}H NMR spectrum of **compound 7**. ^{1}H NMR (400 MHz, CDCl3) δ 8.45 (s, 2H), δ 7.98-7.96 (m, 4H), δ 7.94 (s, 2H) δ 7.81-7.78 (m, 4H), δ 7.64 (s, 2H), 7.25 (t, J = 7.2 Hz, 8H), 7.17 (d, J = 8.4 Hz , H), 2.60 (t, J = 8.0 Hz,8H),1.68-1.60 (m,8H), 1.39-1.27 (m,24H),0.88 (t, J = 6.8 Hz,12H). MS (MADIL-TOF) m/z: [M]$^+$: calcd for $C_{88}H_{82}O_4S_4$; found [M+1]$^+$:1331.635.

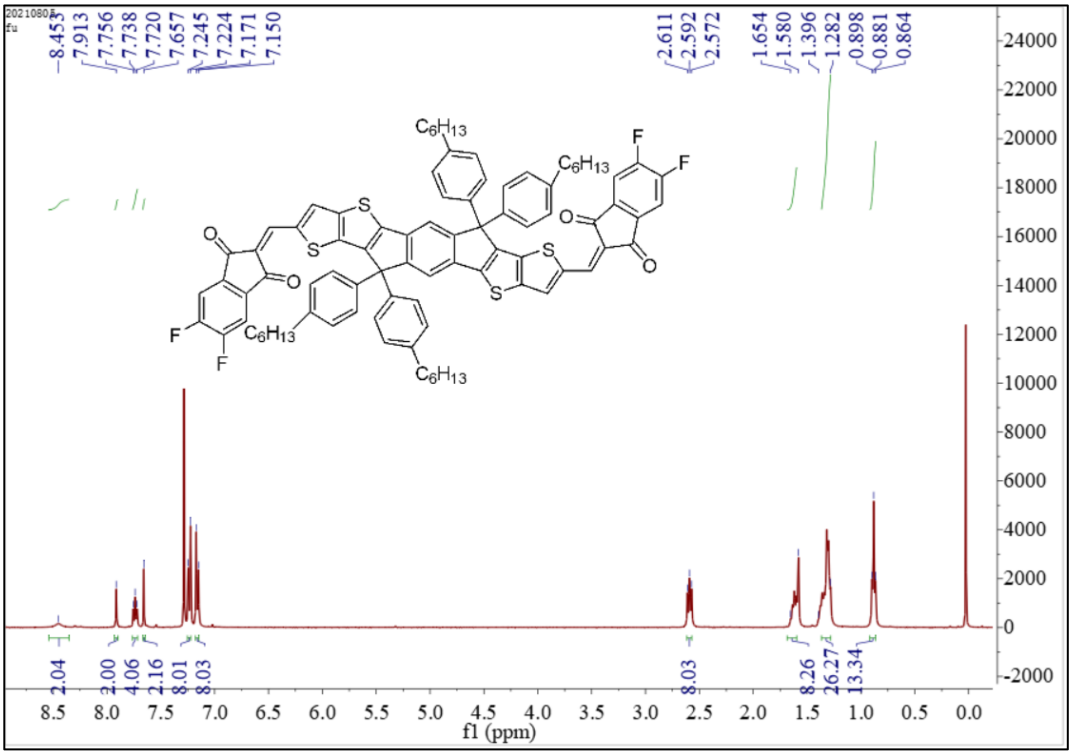

Supplementary Figure 15.^{1}H NMR spectrum of **compound 8**. ^{1}H NMR (400 MHz, $CDCl_3$) δ 8.45 (s, 2H), δ 7.91 (s, 2H), 7.73 (t, J = 7.2 Hz, 4H), 7.66 (s, 2H), 7.24 (d, J = 8.4 Hz ,8H), 7.16 (d, J = 8.4 Hz ,8H), 2.60 (t, J = 8.0 Hz,8H),1.68-1.60 (m,8H), 1.39-1.27 (m,24H),0.88 (t, J = 6.8 Hz,12H). MS (MADIL-TOF) m/z: [M]$^+$: calcd for $C_{88}H_{78}F_4O_4S_4$; found [M+1]$^+$:1403.531.

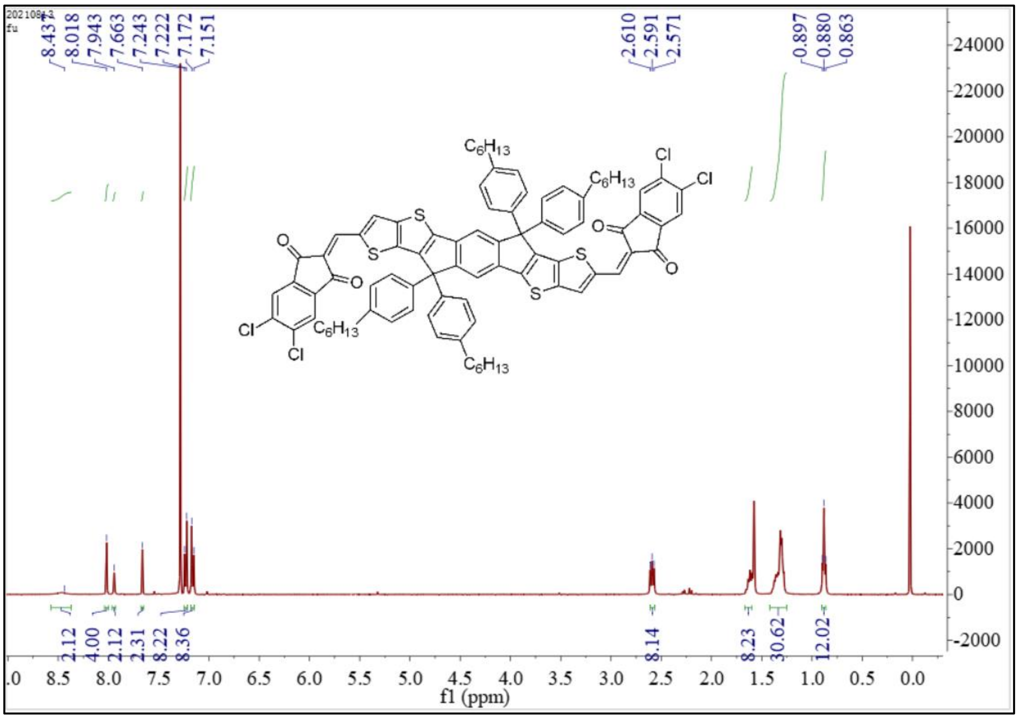

Supplementary Figure 16. ^{1}H NMR spectrum of **compound 9**. ^{1}H NMR (400 MHz, CDCl3) δ 8.02 (s, 4H), δ 7.94 (s, 2H), δ 7.63 (s, 2H), 7.25 (t, J = 7.2 Hz, 8H), 7.17 (d, J = 8.4 Hz , H), 2.60 (t, J = 8.0 Hz,8H),1.68-1.60 (m,8H), 1.39-1.27 (m,24H),0.88 (t, J = 6.8 Hz,12H). MS (MADIL-TOF) m/z: $[M]^+$: calcd for $C_{88}H_{78}Cl_4O_4S_4$; found $[M+1]^+$:1467.481.

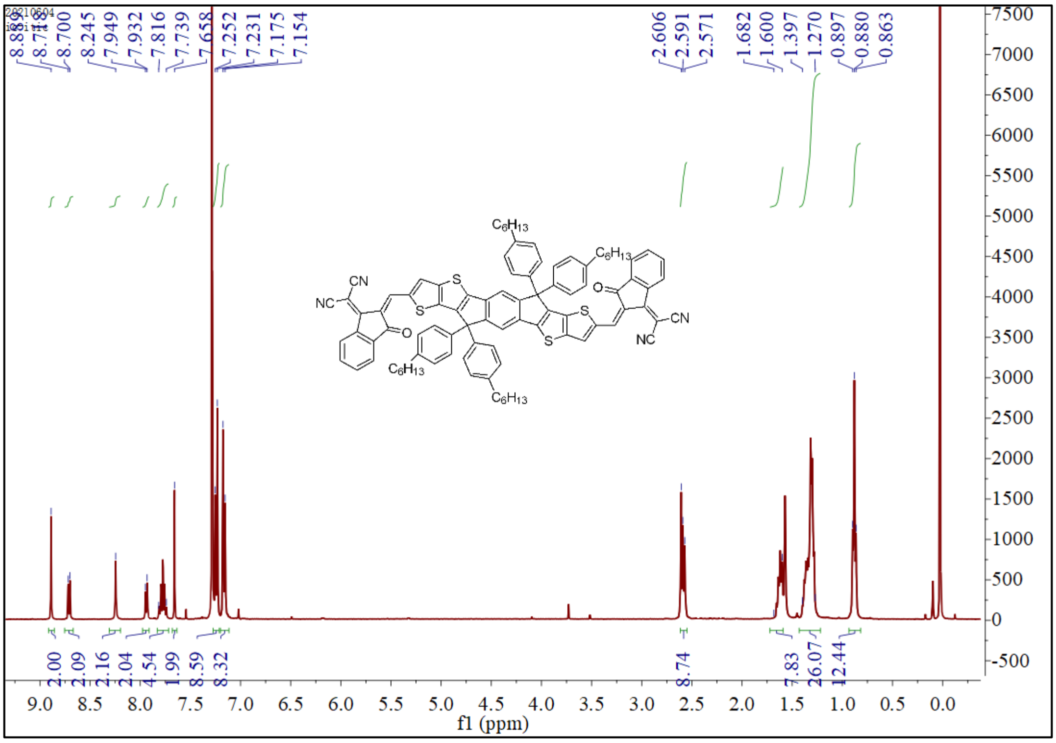

Supplementary Figure 17. ^{1}H NMR spectrum of **compound 10**. ^{1}H NMR (400 MHz, $CDCl_3$) δ 8.89 (s, 2H), 8.71 (d, J = 7.2 Hz, 2H), 8.25 (s, 2H), 7.94 (d, J = 6.8 Hz, 2H),7.81-7.14 (m, 4H), 7.66 (s, 2H), 7.24 (d, J = 8.4 Hz ,4H), 7.16 (d, J = 8.4 Hz ,4H), 2.60 (t, J = 8.0 Hz,8H),1.68-1.60 (m,8H), 1.39-1.27 (m,24H),0.88 (t, J = 6.8 Hz,12H). MS (MADIL-TOF) m/z: $[M]^+$: calcd for $C_{94}H_{82}N_4O_2S_4$; found $[M+1]^+$:1426.635.

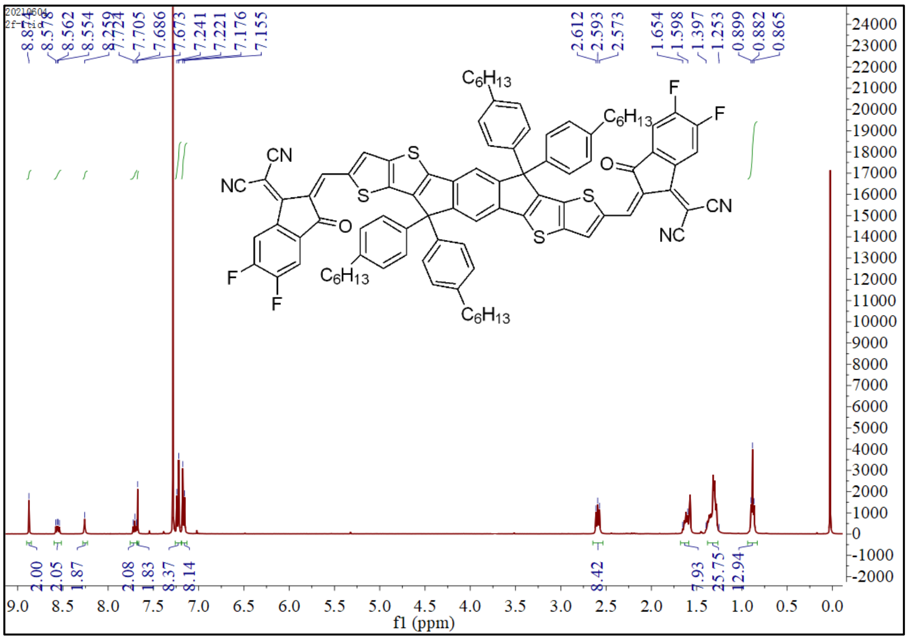

Supplementary Figure 18. ^{1}H NMR spectrum of **compound 11**(IT-4F). ^{1}H NMR (400 MHz, $CDCl_3$) δ 8.87 (s, 2H), 7.45 (dd, J = 6.4 Hz, J = 9.6 Hz, 2H), 8.26 (s, 2H), 7.70 (t, J = 7.6 Hz 2H), 7.67 (s,2H), 7.23 (d, J = 8.0 Hz ,4H), 7.17 (d, J = 8.0 Hz ,4H), 2.56 (t, J = 7.6 Hz,8H),1.65-1.59 (m,8H), 1.40-1.25 (m,24H),0.88 (t, J = 6.8 Hz,12H). MS (MADIL-TOF) m/z: $[M]^+$: calcd for $C_{94}H_{78}F_4N_4O_2S_4$; found $[M+1]^+$:1499.554.

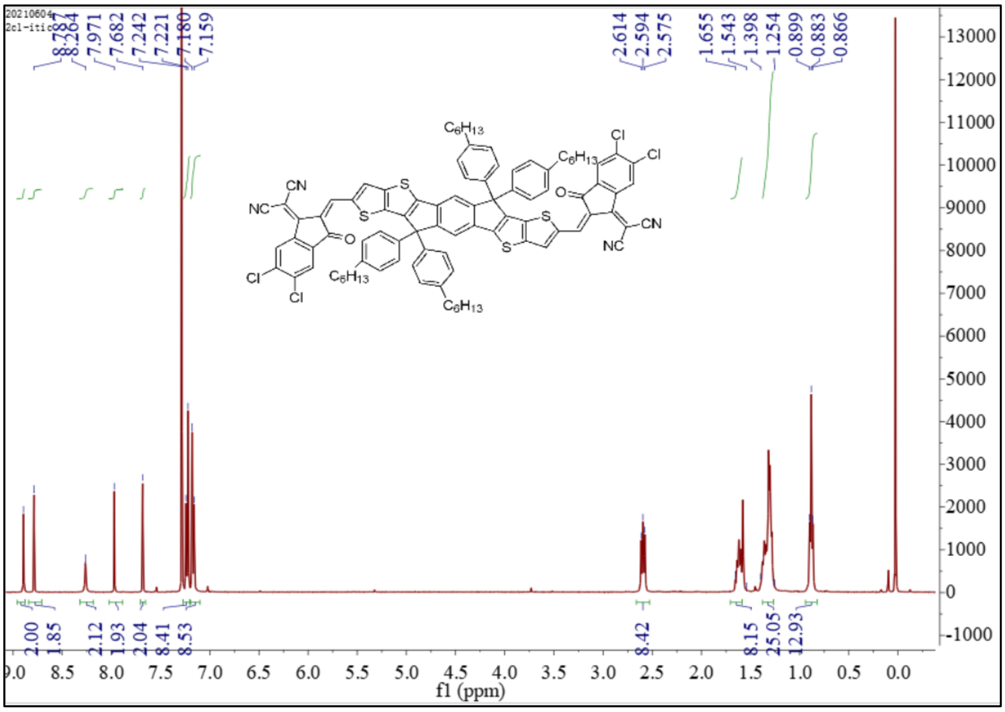

Supplementary Figure 19. ^{1}H NMR spectrum of **compound 12**. ^{1}H NMR (400 MHz, $CDCl_3$) δ 8.90 (s, 2H), 8.79 (s, 2H), 8.26 (s, 2H), 7.97 (s, 2H),7.68 (s, 2H), 7.23 (d, J = 8.0 Hz ,4H), 7.17 (d, J = 8.0 Hz ,4H), 2.60 (t, J = 7.6 Hz,8H),1.65-1.54 (m,8H), 1.39-1.25 (m,24H),0.88 (t, J = 6.8 Hz,12H). MS (MADIL-TOF) m/z: $[M]^+$: calcd for $C_{94}H_{78}Cl_4N_4O_2S_4$; found $[M+1]^+$:1563.421.

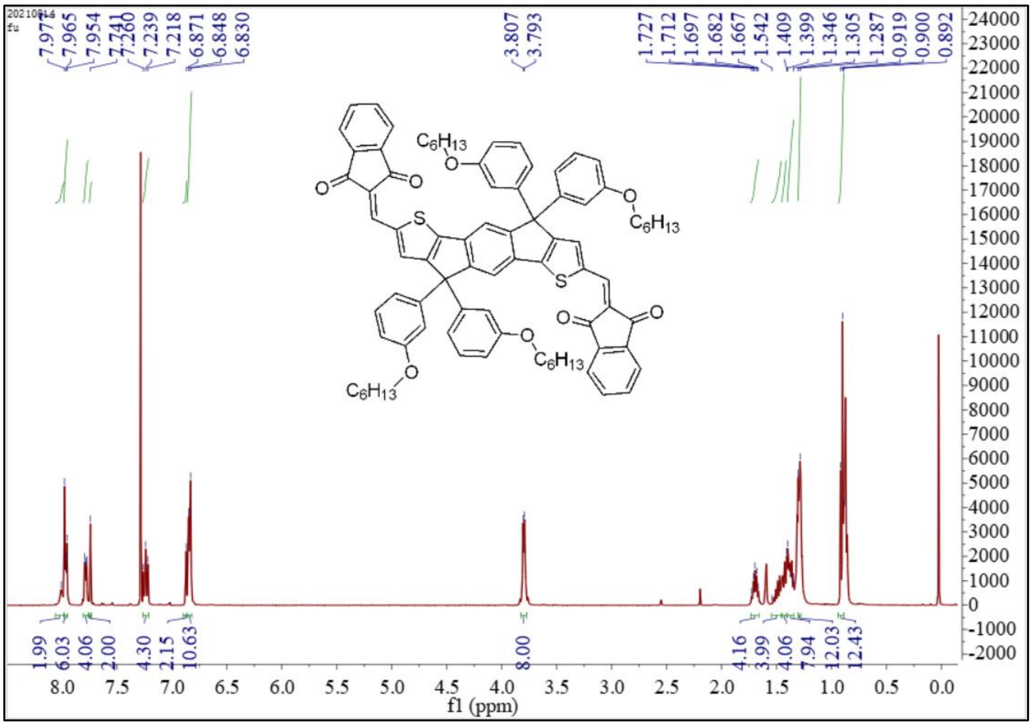

Supplementary Figure 20. ^{1}H NMR spectrum of **compound 13**. ^{1}H NMR (400 MHz, CDCl3) δ 8.01 (s, 2H), 7.98-7.95 (m, 6H), 7.80-7.78 (m, 4H), 7.74 (s, 2H), 7.24 (t, J = 8.0 Hz,4H), 6.87(s,2H), 6.85-6.83 (m, 10H), 6.79 (s, 4H), 3.80(d, J = 5.2 Hz, 8H), 1.74-1.65 (m,4H), 1.52-1.46 (m,4H), 1.44-1.40 (m,4H),1.40-1.36 (m,8H),1.31-1.28(m,12H), 0.90 (t, J = 6.8 Hz, 12H) MS (MADIL-TOF) m/z: [M]$^+$: calcd for $C_{84}H_{82}O_8S_2$; found [M+1]$^+$:1283.527.

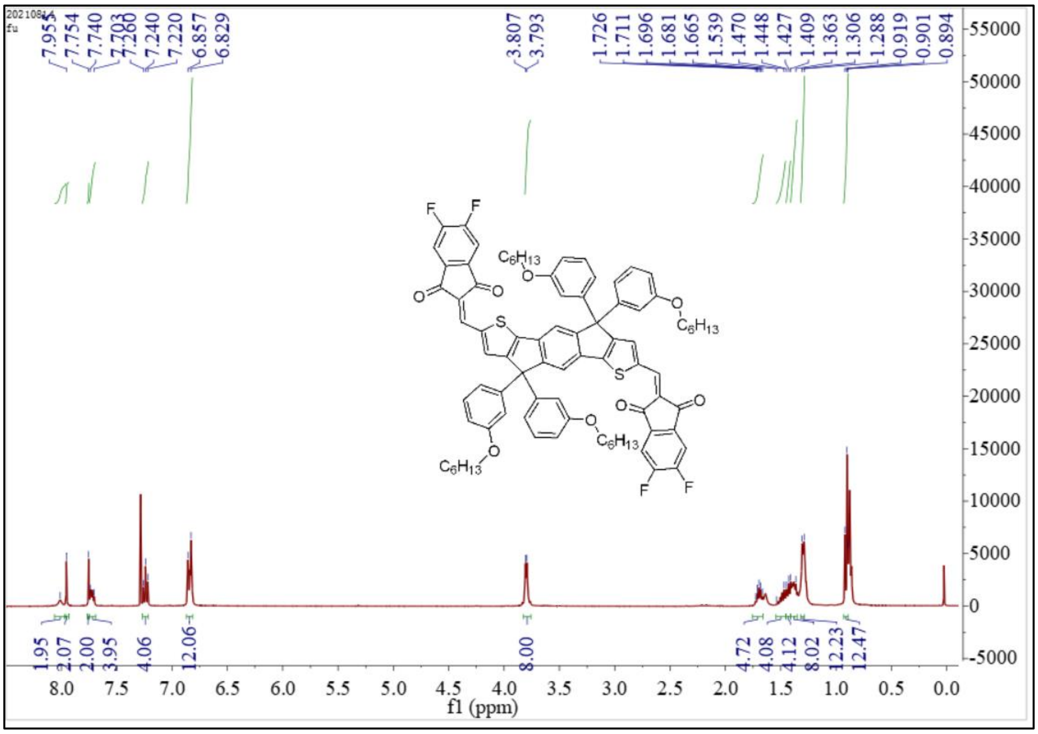

Supplementary Figure 21. ^{1}H NMR spectrum of **compound 14**. ^{1}H NMR (400 MHz, CDCl3) δ 8.01 (s, 2H), 7.96 (s, 2H), 7.75 (s, 2H), 7.74-7.70 (m, 4H), 7.24 (t, J = 8.0 Hz,4H), 6.86-6.83(m,12H), 3.80(d, J = 5.2 Hz, 8H), 1.74-1.65 (m,4H), 1.52-1.46 (m,4H), 1.44-1.40 (m,4H),1.40-1.36

(m,8H),1.31-1.28(m,12H), 0.90 (t, J = 6.8 Hz, 12H) MS (MADIL-TOF) m/z: $[M]^+$: calcd for $C_{84}H_{78}F_4O_8S_2$; found $[M+1]^+$:1355.580.

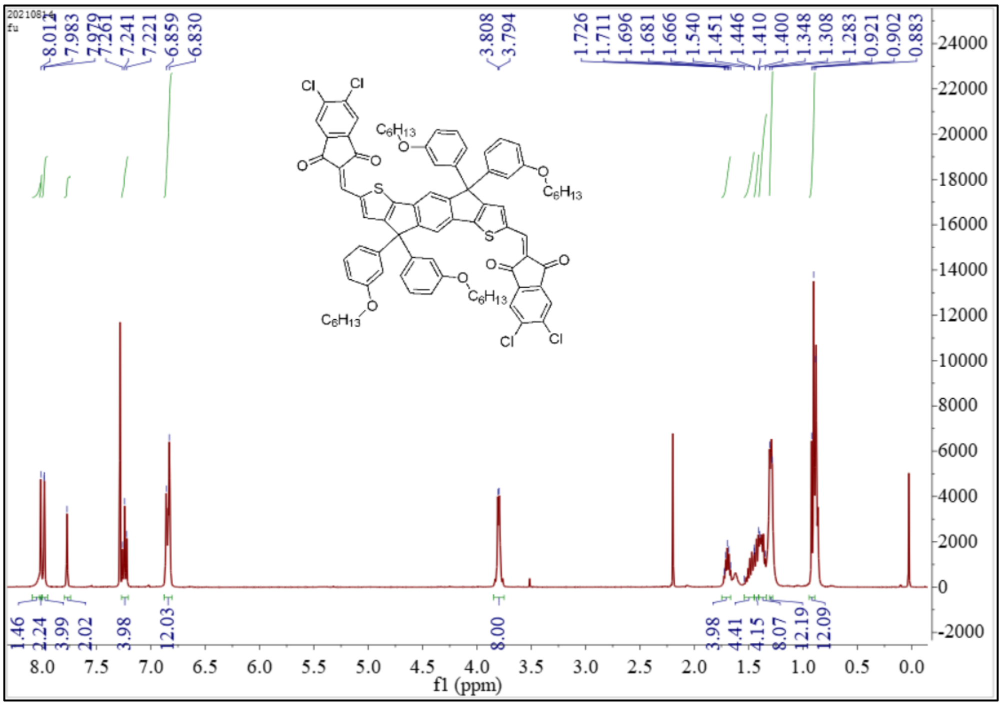

Supplementary Figure 22. ^{1}H NMR spectrum of **compound 15**. ^{1}H NMR (400 MHz, $CDCl_3$) δ 8.01 (s, 2H), 7.98 (s, 2H), 7.97 (s, 4H), 7.77 (s, 2H), 7.24 (t, J = 8.0 Hz,4H), 6.86-6.83(m,12H), 3.80(d, J = 5.2 Hz, 8H), 1.74-1.65 (m,4H), 1.52-1.46 (m,4H), 1.44-1.40 (m,4H),1.40-1.36 (m,8H),1.31-1.28(m,12H), 0.90 (t, J = 6.8 Hz, 12H) MS (MADIL-TOF) m/z: $[M]^+$: calcd for $C_{84}H_{78}Cl_4O_8S_2$; found $[M+1]^+$:1419.428.

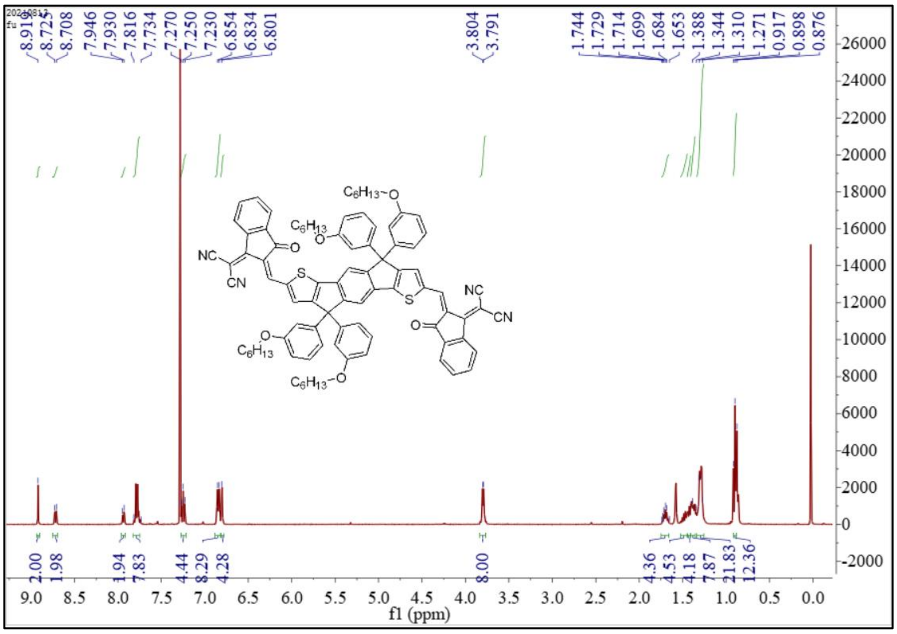

Supplementary Figure 23. ^{1}H NMR spectrum of **compound 16**. 8.92 (s, 2H), 8.71 (d, J = 6.8 Hz, 2H), 7.94 (d, J = 6.8 Hz, 2H), 7.82-7.73 (m,8H), 7.25 (t, J = 8.0 Hz, 4H), 6.84 (d, J = 8.0 Hz, 8H), 3.80(d, J = 5.2 Hz, 8H), 1.74-1.65 (m,4H), 1.52-1.46 (m,4H), 1.44-1.40 (m,4H),1.40-1.36

(m,8H),1.31-1.28(m,12H), 0.90 (t, J = 6.8 Hz, 12H) MS (MADIL-TOF) m/z: $[M]^+$: calcd for $C_{90}H_{82}N_4O_6S_2$; found $[M+1]^+$:1379.611.

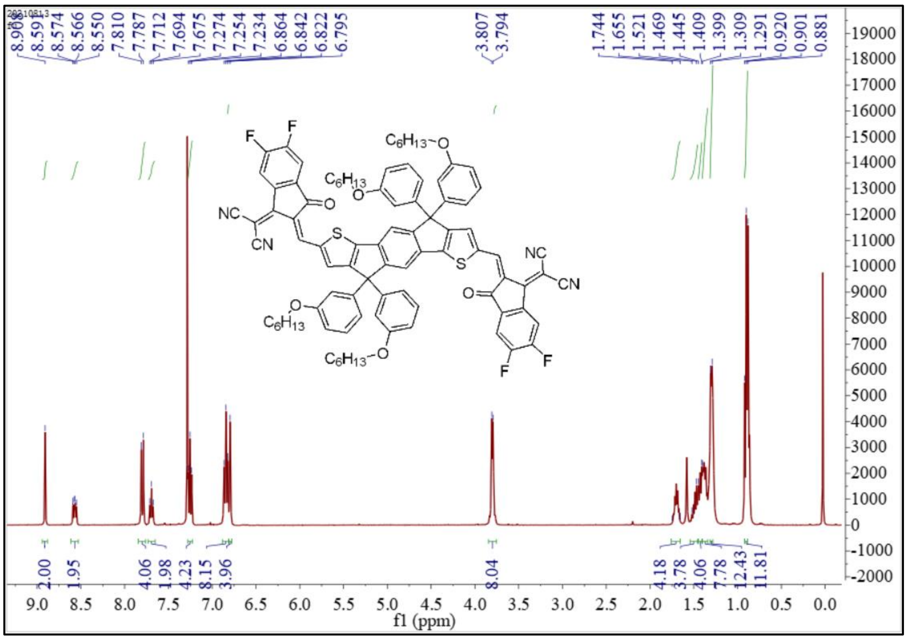

Supplementary Figure 24. ^{1}H NMR spectrum of **compound 17**. ^{1}H NMR (400 MHz, CDCl3) δ 8.90 (s, 2H), 8.56 (dd, J = 6.8 Hz, J = 10.0 Hz, 2H), 7.80 (d, J = 9.2 Hz, 4H), 7.69 (t, J = 7.6 Hz, 2H), 7.25 (t, J = 8.0 Hz,4H), 6.84 (t, J = 8.0 Hz, 8H), 6.79 (s, 4H), 3.80(d, J = 5.2 Hz, 8H), 1.74-1.65 (m,4H), 1.52-1.46 (m,4H), 1.44-1.40 (m,4H),1.40-1.36 (m,8H),1.31-1.28(m,12H), 0.90 (t, J = 6.8 Hz, 12H) MS (MADIL-TOF) m/z: $[M]^+$: calcd for $C_{90}H_{78}F_4N_4O_6S_2$; found $[M+1]^+$:1451.592.

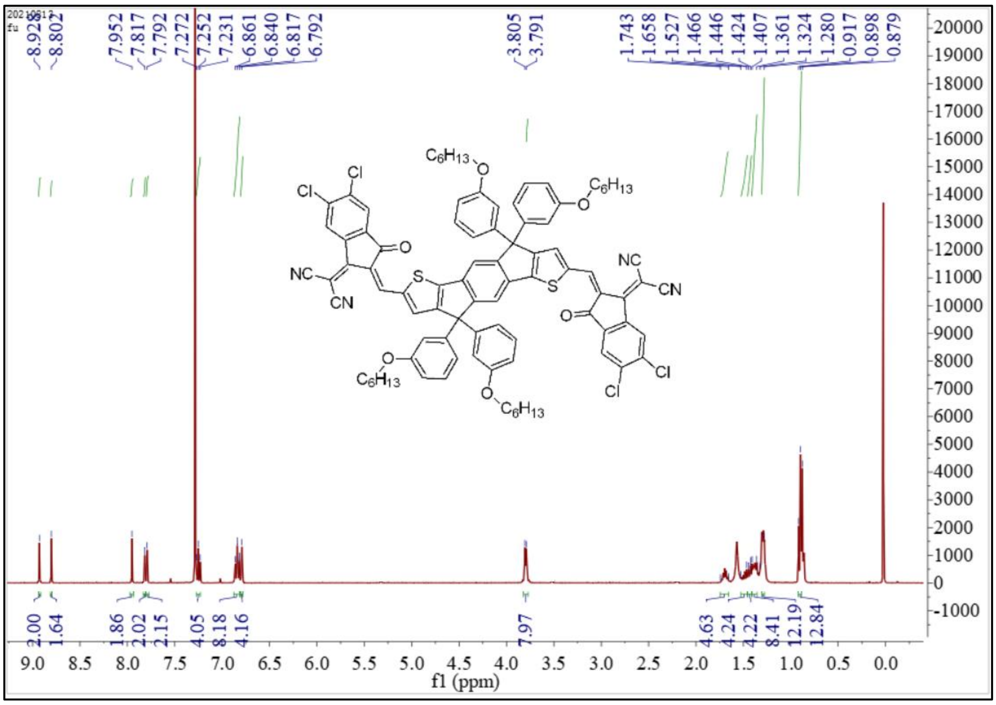

Supplementary Figure 25. ^{1}H NMR spectrum of **compound 18**. ^{1}H NMR (400 MHz, CDCl3) δ 8.92 (s, 2H), 8.80 (s, 2H), 7.95 (s, 4H), 7.81 (s, 2H), 7.79 (s, 2H), 7.25 (t, J = 8.0 Hz,4H), 6.84 (t, J = 8.0 Hz, 8H), 6.79 (s, 4H), 3.80(d, J = 5.2 Hz, 8H), 1.74-1.65 (m,4H), 1.52-1.46 (m,4H), 1.44-1.40 (m,4H),1.40-1.36 (m,8H),1.31-1.28(m,12H), 0.90 (t, J = 6.8 Hz, 12H) MS (MADIL-TOF) m/z: $[M]^+$: calcd for $C_{90}H_{78}Cl_4N_4O_6S_2$; found $[M+1]^+$:1515.610.

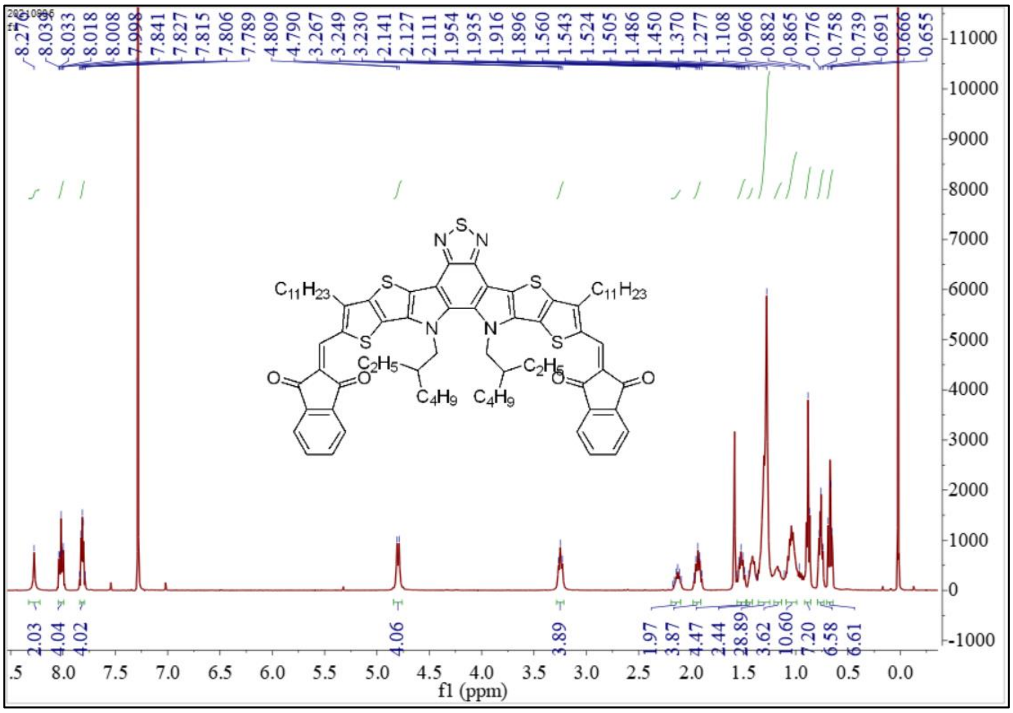

Supplementary Figure 26. ^{1}H NMR spectrum of **compound 19**. ^{1}H NMR (400 MHz, CDCl3) δ 8.28 (s, 2H), 8.04-8.00 (m, 4H), 8.02-7.79 (m, 2H),7.84-7.79(m, 4H) 4.80(d, J = 6.0 Hz, 4H), 3.25(t, J = 7.6 Hz, 4H), 2.21-2.12 (m,2H), 1.94-1.86 (m,4H),1.35-1.15 (m,36H),1.15-0.99(m,12H), 0.88(t, J = 6.8 Hz, 6H), 0.80(t, J = 4.8 Hz, 6H) , 0.70(t, J = 7.2 Hz, 6H). MS (MADIL-TOF) m/z: [M]$^+$: calcd for $C_{76}H_{90}N_4O_4S_5$; found [M+1]$^+$:1283.634.

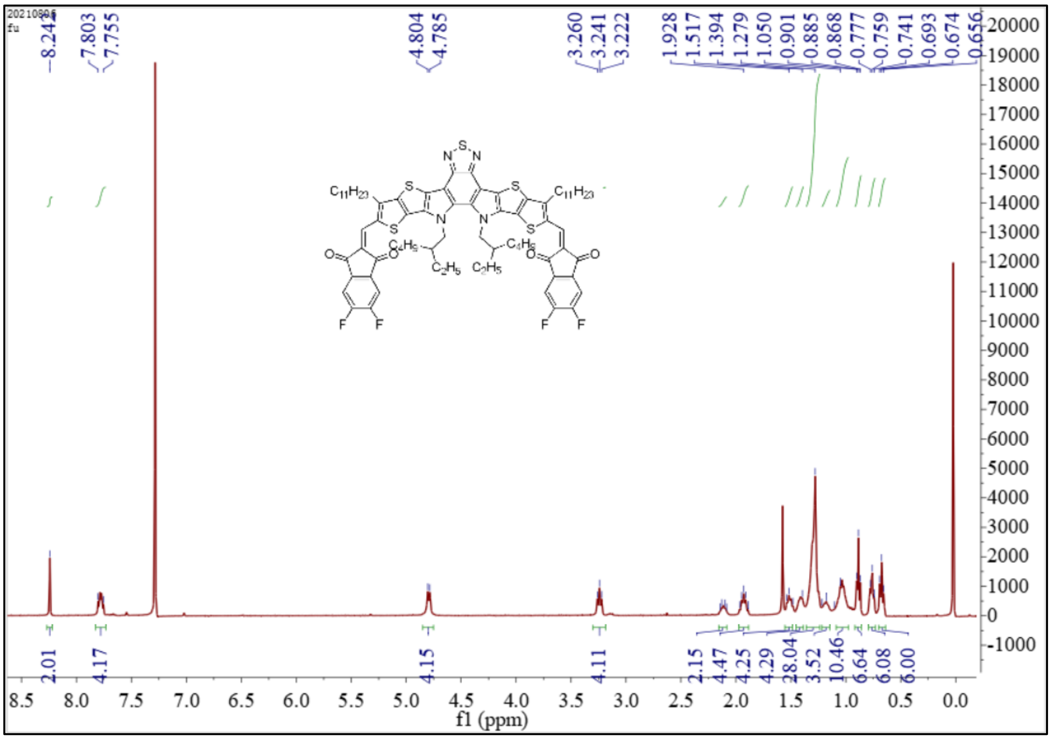

Supplementary Figure 27. ^{1}H NMR spectrum of **compound 20**. ^{1}H NMR (400 MHz, CDCl3) δ 8.24 (s, 2H), 7.80-7.76 (m, 4H), 4.80(d, J = 6.0 Hz, 4H), 3.25(t, J = 7.6 Hz, 4H), 2.21-2.12 (m,2H), 1.94-1.86 (m,4H),1.35-1.15 (m,36H),1.15-0.99(m,12H), 0.88(t, J = 6.8 Hz, 6H), 0.80(t, J = 4.8 Hz, 6H) ,

0.70(t, J = 7.2 Hz, 6H). MS (MADIL-TOF) m/z: $[M]^+$: calcd for $C_{76}H_{86}F_4N_4O_4S_5$; found $[M+1]^+$:1355.627.

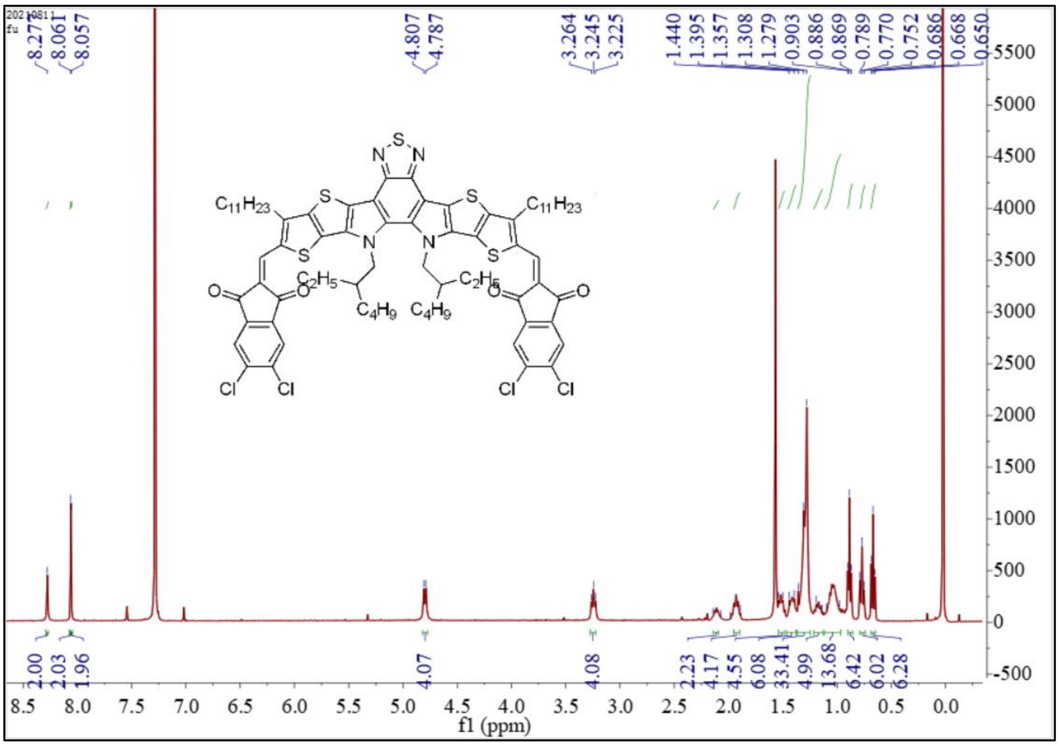

Supplementary Figure 28. ^{1}H NMR spectrum of **compound 21**. ^{1}H NMR (400 MHz, CDCl3) δ 8.27 (s, 2H), 8.06 (s, 2H), 8.05 (s, 2H), 4.79(d, J = 6.0 Hz, 4H), 3.25(t, J = 7.6 Hz, 4H), 2.21-2.12 (m,2H), 1.94-1.86 (m,4H),1.35-1.15 (m,36H),1.15-0.99(m,12H), 0.88(t, J = 6.8 Hz, 6H), 0.80(t, J = 4.8 Hz, 6H) , 0.70(t, J = 7.2 Hz, 6H). MS (MADIL-TOF) m/z: $[M]^+$: calcd for $C_{76}H_{86}Cl_4N_4O_4S_5$; found $[M+1]^+$:1419.627.

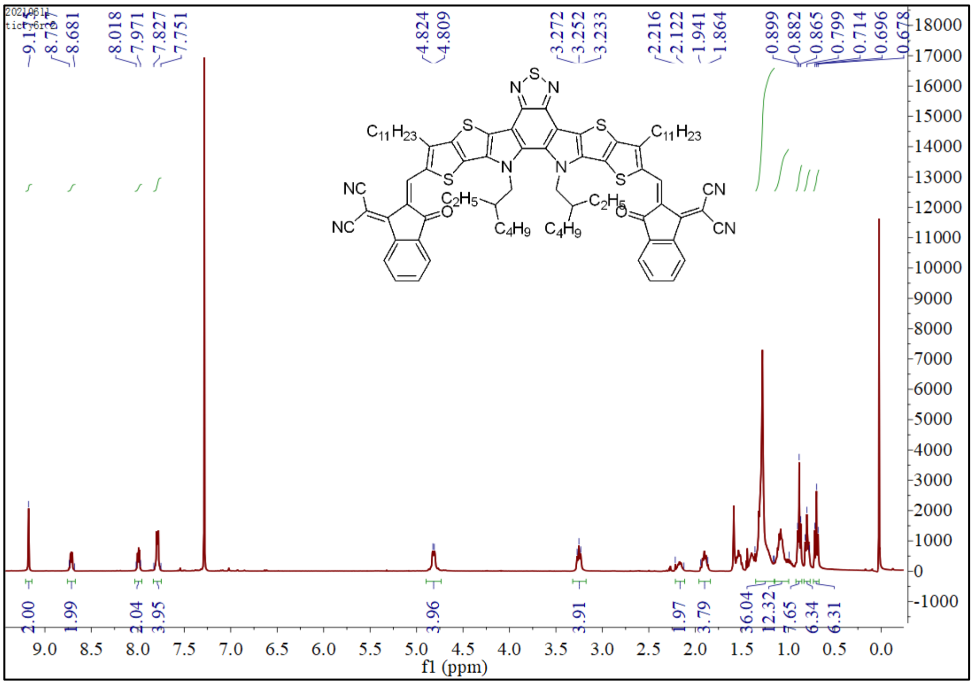

Supplementary Figure 29. ^{1}H NMR spectrum of **compound 22**(Y5-2BO). ^{1}H NMR (400 MHz, CDCl3) δ 9.18 (s, 2H), 8.74-8.68 (m, 2H), 8.02-7.79 (m, 2H),7.83-7.79(m, 4H) 4.82(d, J = 6.0 Hz, 4H), 3.25(t, J = 7.6 Hz, 4H), 2.21-2.12 (m,2H), 1.94-1.86 (m,4H),1.35-1.15 (m,36H),1.15-

0.99(m,12H), 0.88(t, J = 6.8 Hz, 6H), 0.80(t, J = 4.8 Hz, 6H) , 0.70(t, J = 7.2 Hz, 6H). MS (MADIL-TOF) m/z: [M]$^+$: calcd for $C_{82}H_{90}N_8O_2S_5$; found [M+1]$^+$:1379.625.

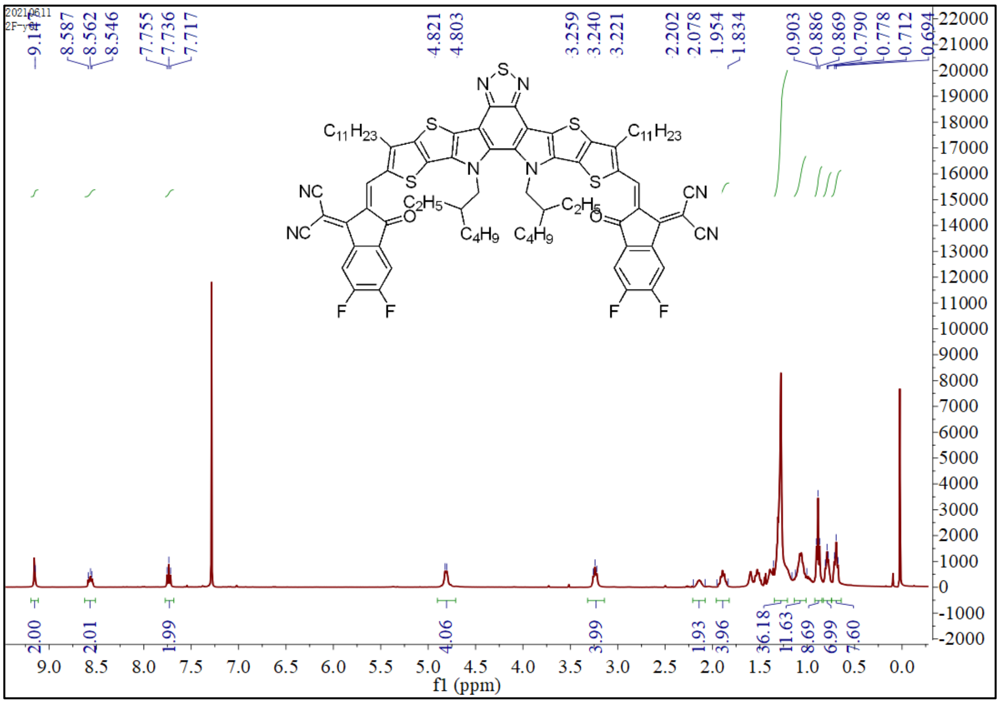

Supplementary Figure 30. ^{1}H NMR spectrum of **compound 23**(Y6). ^{1}H NMR (400 MHz, CDCl3) δ 9.15 (s, 2H), 8.56 (t, J = 6.4 Hz, 2H), 7.73 (t, J = 7.6 Hz, 2H), 4.81(d, J = 7.2 Hz, 4H), 3.24(d, J = 7.6 Hz, 4H), 2.20-2.01 (m,2H), 1.95-1.83 (m,4H),1.36-1.16 (m,36H),1.12-1.00(m,12H), 0.87(t, J = 6.8 Hz, 6H), 0.79(t, J = 4.8 Hz, 6H) , 0.69(t, J = 7.2 Hz, 6H). MS (MADIL-TOF) m/z: [M]$^+$: calcd for $C_{82}H_{86}F_4N_8O_2S_5$; found [M+1]$^+$:1451.589.

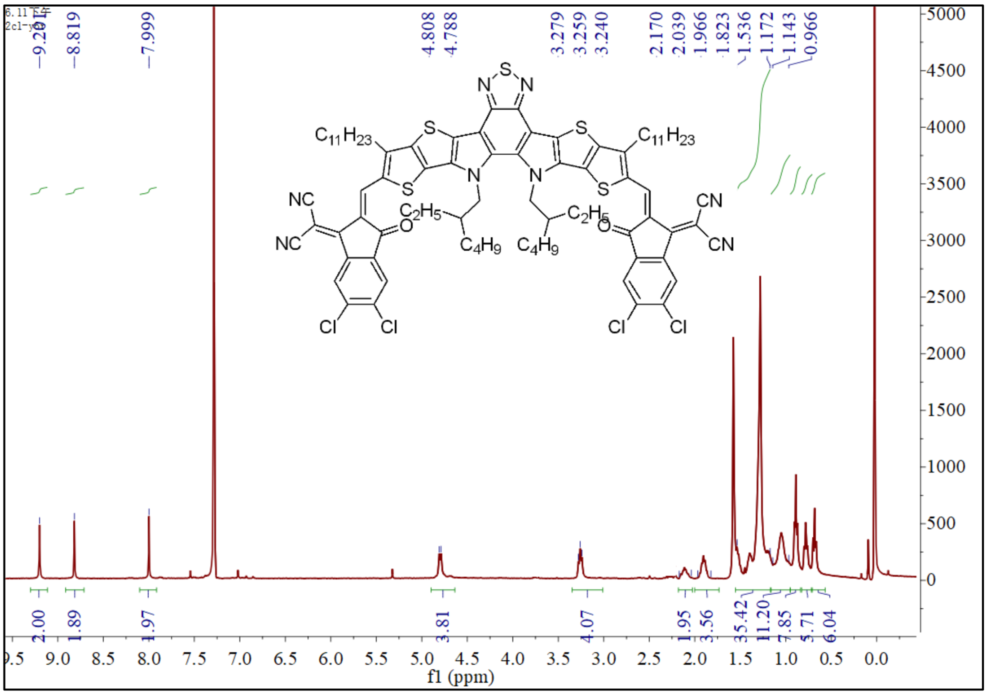

Supplementary Figure 31. ^{1}H NMR spectrum of **compound 24**(BTIC-BO-4C). ^{1}H NMR (400 MHz, CDCl3) δ 9.20 (s, 2H), 8.82 (s, 2H), 8.00 (s, 2H), 4.80(d, J = 8.0 Hz, 4H), 3.26(d, J = 7.6 Hz, 4H), 2.17-2.04 (m,2H), 1.96-1.82 (m,4H),1.36-1.16 (m,36H),1.12-0.96(m,12H), 0.87(t, J = 6.8 Hz, 6H),

0.79(t, J = 4.8 Hz, 6H) , 0.69(t, J = 7.2 Hz, 6H). MS (MADIL-TOF) m/z: $[M]^+$: calcd for $C_{82}H_{86}Cl_4N_8O_2S_5$; found $[M+1]^+$:1515.538.

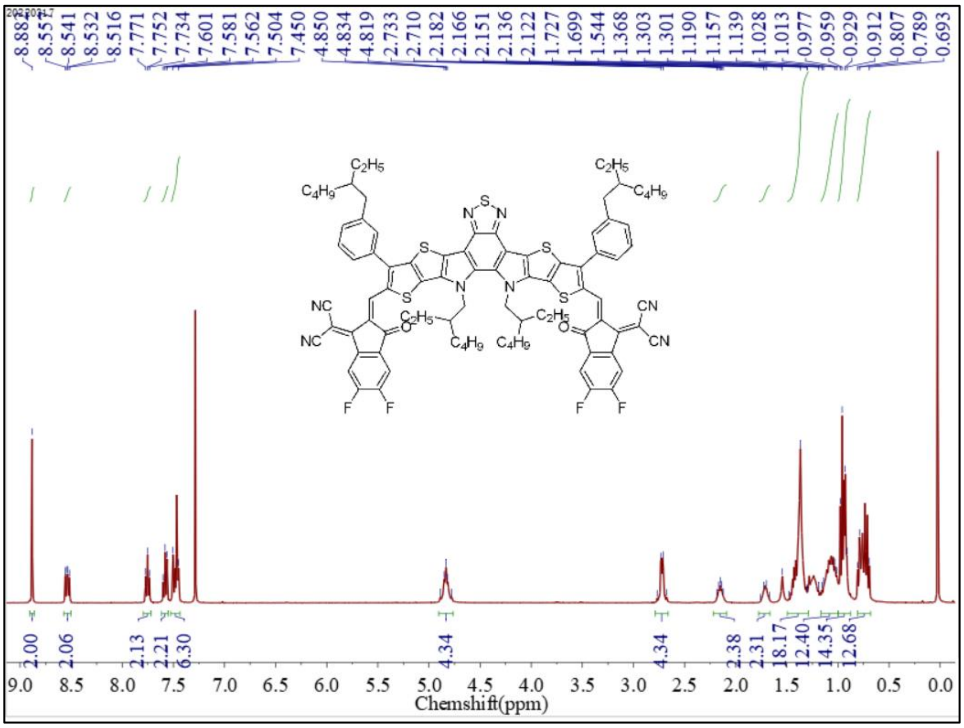

Supplementary Figure 32. ^{1}H NMR spectrum of **compound 25(**BTP-4F-P2EH). ^{1}H NMR (400 MHz, $CDCl_3$) δ 8.88 (s, 2H), 8.53 (dd, J = 6.4 Hz,10.0 Hz, 2H), 7.75 (t, J = 7.2 Hz, 2H),7.58(t, J=7.6Hz),7.50-7.45(m,6H),4.88-4.77(m, 4H), 2.76-2.67(m, 4H), 2.18-2.12 (m,2H), 1.75-1.67 (m,2H),1.40-1.30 (m,18H),1.15-1.01(m,12H), 0.97-0.91(m, 14H), 0.80-0.69(m, 12H). MS (MADIL-TOF) m/z: $[M]^+$: calcd for $C_{88}H_{82}F_4N_8O_2S_5$; found $[M+1]^+$:1519.689

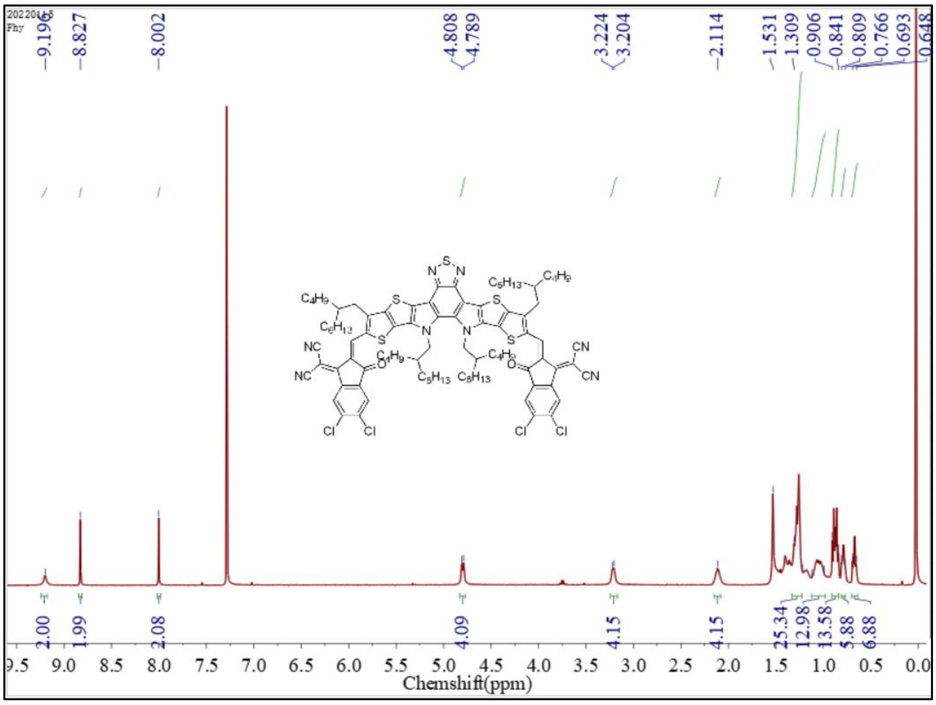

Supplementary Figure 33. ^{1}H NMR spectrum of **compound 26**. ^{1}H NMR (400 MHz, $CDCl_3$) δ 9.19 (s, 2H), 8.82 (S, 2H), 8.00(s, 2H),4.79(d, J = 7.6 Hz 4H), 3.21(d, J = 8.0 Hz, 4H), 2.11(m, 4H), 2.21-

2.12 (m,2H), 1.30-1.21 (m,24H), 1.11-0.99(m,12H), 0.90-0.84(m, 12H), 0.80-0.76(m, 6H), 0.69-0.64(m, 6H). MS (MADIL-TOF) m/z: $[M]^+$: calcd for $C_{84}H_{90}Cl_4N_8O_2S_5$; found $[M+1]^+$:1543.521.

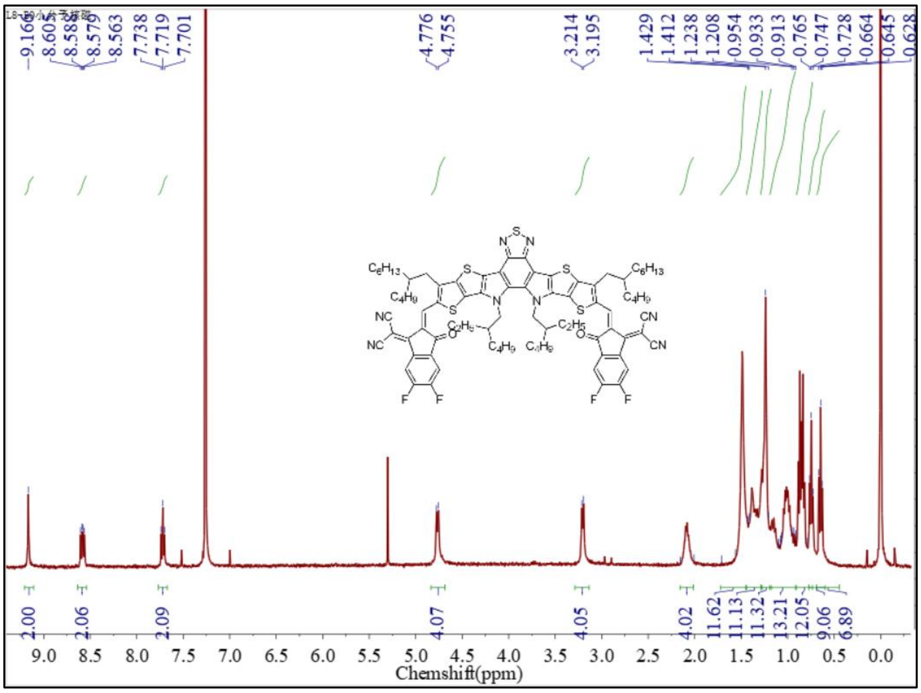

Supplementary Figure 34. ^{1}H NMR spectrum of **compound 27(**L8-BO). ^{1}H NMR (400 MHz, $CDCl_3$) δ 9.16 (s, 2H), 8.58 (dd, J = 6.8 Hz,10.4 Hz, 2H), 7.72 (t, J = 7.2 Hz, 2H), 4.76(d, , J = 8.4 Hz,4H), 3.20(d, J = 7.6 Hz, 4H),2.00(m,4H), 1.70-1.42 (m,12H),1.41-1.27 (m,12H),1.20-1.08(m,12H), 0.90-0.81(m, 12H), 0.74(t, J= 7.6Hz,6H), 0.64(t, J= 7.6Hz,6H). MS (MADIL-TOF) m/z: $[M]^+$: calcd for $C_{84}H_{90}F_4N_8O_2S_5$; found $[M+1]^+$:1479.610

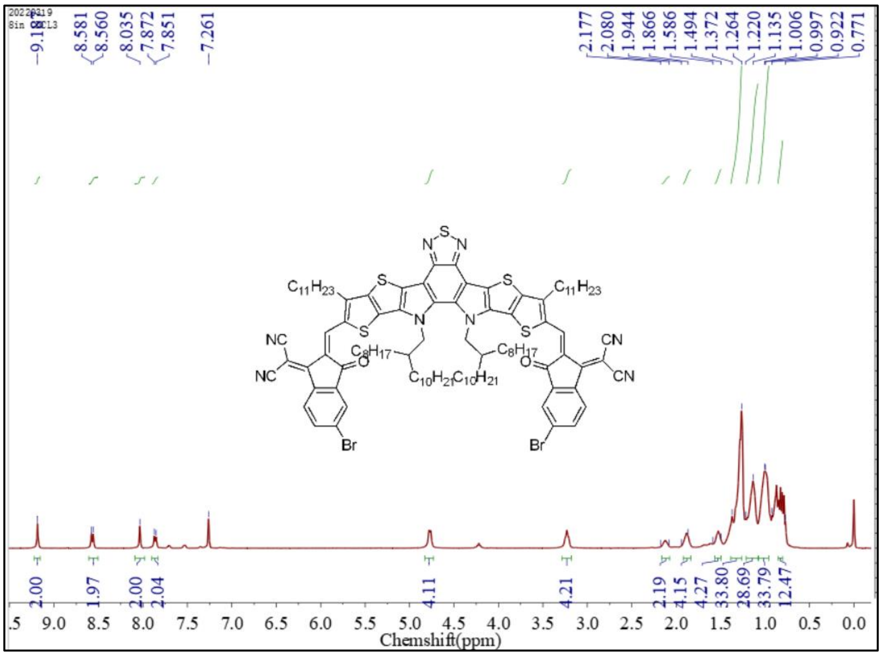

Supplementary Figure 35. ^{1}H NMR spectrum of **compound 28(**Y5-I-C20-Br). ^{1}H NMR (400 MHz, $CDCl_3$) δ 9.18 (s, 2H), 8.57 (d, *J* = 8.0 Hz, 2H), 8.03 (s, 2H), 7.86 (d, *J* = 7.9 Hz, 2H), 4.78 (d, *J* = 7.5 Hz, 4H), 3.21 (t, *J* = 7.6 Hz, 4H), 2.20-2.09 (m, 2H), 1.94-1.83 (m,4H), 1.61-1.46(m, 4H), 1.33 – 1.21 (m, 32H), 1.21 – 1.12 (m, 28H), 1.10-1.98 (m,32H), 0.88 – 0.72 (m, 12H). MS (MADIL-TOF) m/z: [M]+: calcd for $C_{106}H_{136}Br_2N_8O_2S_5$; found [M+1]+:1871.811

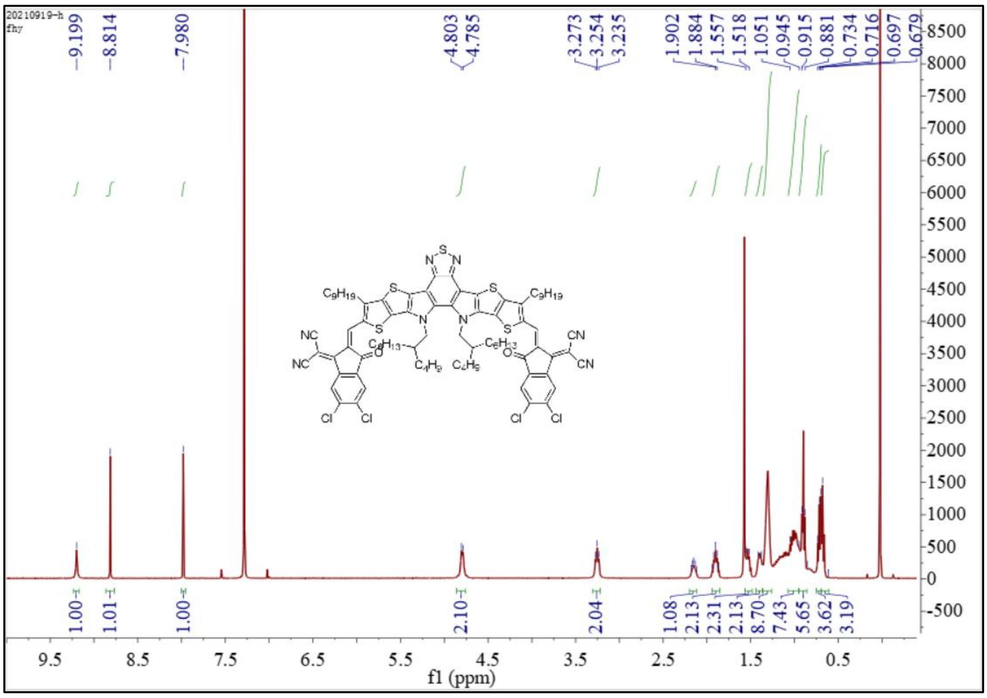

Supplementary Figure 36. ^{1}H NMR spectrum of **compound 29(**BTP-eC9). ^{1}H NMR (400 MHz, $CDCl_3$) δ 9.19 (s, 2H), 8.81 (s, 2H), 7.98 (s, 2H), 4.79(d, J= 8.0 Hz, 4H), 3.25(d, J = 7.6 Hz, 4H), 2.17-2.04 (m,2H), 1.96-1.82 (m,4H),1.36-1.16 (m,44H),1.12-0.96(m,12H), 0.87(t, J = 6.8 Hz, 6H), 0.79(t, J = 4.8 Hz, 6H) , 0.69(t, J = 7.2 Hz, 6H). MS (MADIL-TOF) m/z: $[M]^+$: calcd for $C_{86}H_{94}Cl_4N_8O_2S_5$; found $[M+1]^+$:1571.548.

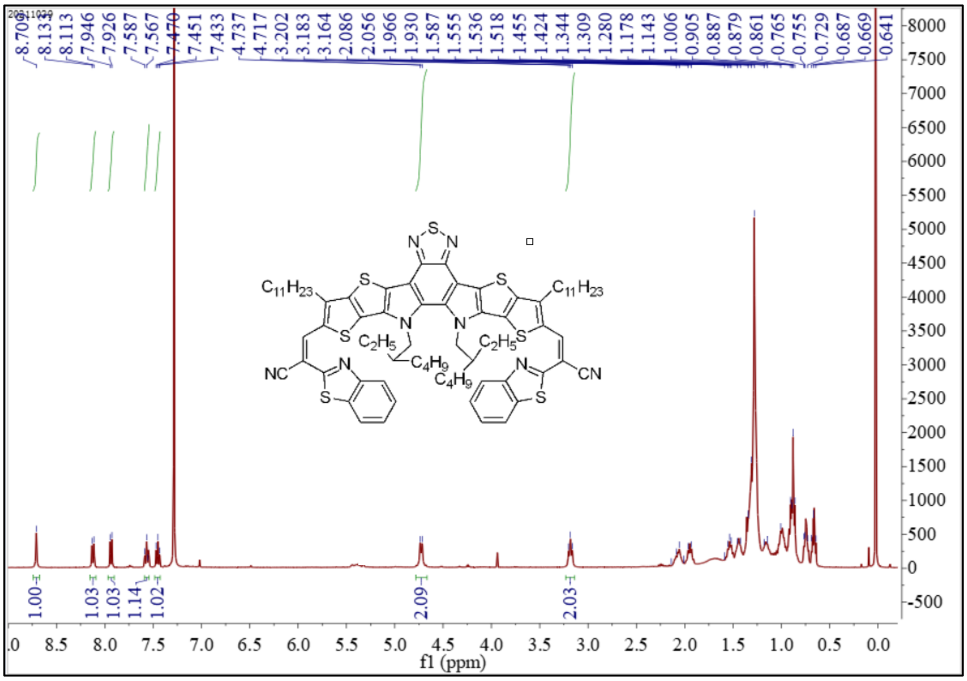

Supplementary Figure 37. ^{1}H NMR spectrum of **compound 30**. ^{1}H NMR (400 MHz, $CDCl_3$) δ 8.70 (s, 2H), 8.12 (d, J = 8.0 Hz, 2H), 7.93 (d, J = 8.0 Hz, 2H), 7.56 (t, J = 7.2 Hz, 2H), 7.45 (t, J = 7.2 Hz, 2H), 4.72(d, J= 8.0 Hz, 4H), 3.18(d, J = 7.6 Hz, 4H), 2.17-2.04 (m,2H), 1.96-1.82

(m,4H),1.36-1.16 (m,44H),1.12-0.96(m,12H), 0.87(t, J = 6.8 Hz, 6H), 0.79(t, J = 4.8 Hz, 6H) , 0.69(t, J = 7.2 Hz, 6H). MS (MADIL-TOF) m/z: $[M]^+$: calcd for $C_{76}H_{90}Cl_4N_8S_7$; found $[M+1]^+$:1339.548.

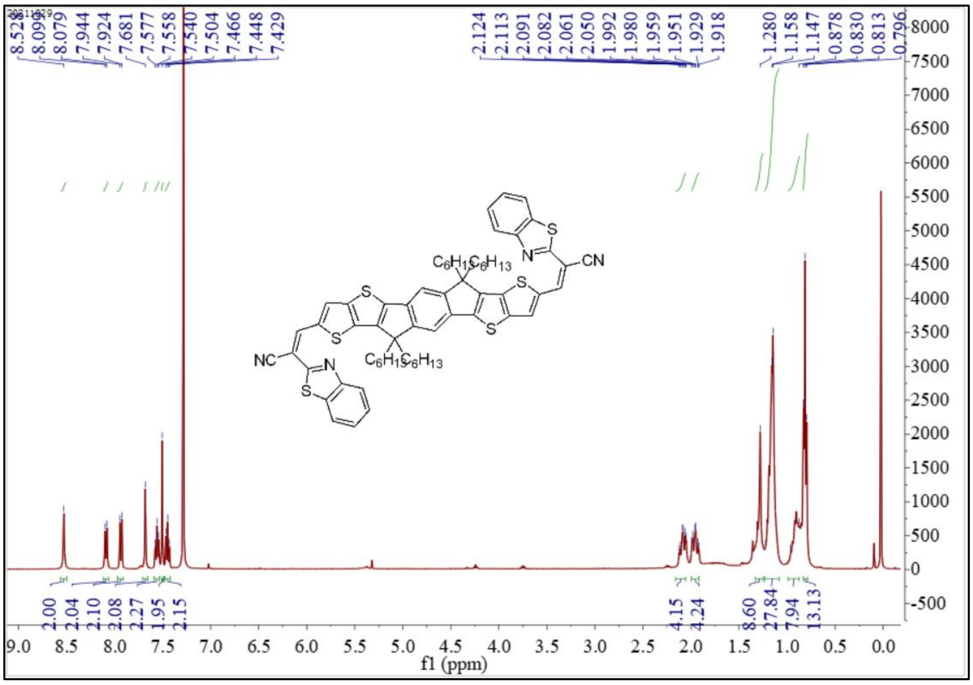

Supplementary Figure 38. ^{1}H NMR spectrum of **compound 31**.^{1}H NMR (400 MHz, $CDCl_3$) δ 8.52 (d, 2H), 8.10 (d, J = 8.0 Hz, 2H), 7.93 (d, J = 8.0 Hz, 2H), 7.68 (s,2H), 7.55 (t, J = 7.6 Hz, 2H), 7.50 (s,2H), 7.44 (t, J = 7.6 Hz, 2H),2.12 (dt, J = 3.6 Hz, J = 13.6 Hz, 4H), 1.95 (dt, J = 3.6 Hz, J = 13.6 Hz, 4H).1.24-1.06 (m,24H), 0.98-0.85 (m,8H),0.81 (t, J = 7.2 Hz,12H). MS (MADIL-TOF) m/z: $[M]^+$: calcd for $C_{60}H_{66}N_4S_4$; found $[M+1]^+$: 971.437.

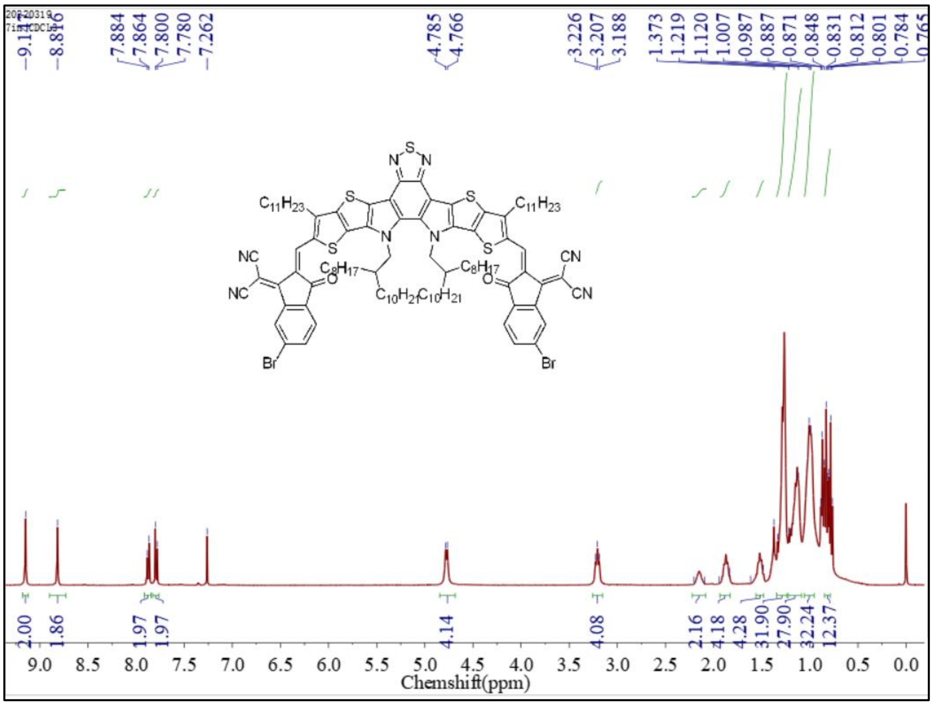

Supplementary Figure 39. ^{1}H NMR spectrum of **compound 32**(Y5-O-C20-Br).^{1}H NMR (400 MHz, $CDCl_3$) δ 9.15 (s, 2H), 8.82 (s, 2H), 7.87 (d, *J* = 8.0 Hz, 2H), 7.79 (d, *J* = 7.9 Hz, 2H), 4.78 (d, *J* = 7.5 Hz, 4H), 3.21 (t, *J* = 7.6 Hz, 4H), 2.20-2.09 (m, 2H), 1.94-1.83 (m,4H), 1.61-1.46(m, 4H), 1.33 –

1.21 (m, 32H), 1.21 – 1.12 (m, 28H), 1.10-1.98 (m,32H), 0.88 – 0.72 (m, 12H). MS (MADIL-TOF) m/z: [M]+: calcd for $C_{106}H_{136}Br_2N_8O_2S_5$; found [M+1]+:1871.803

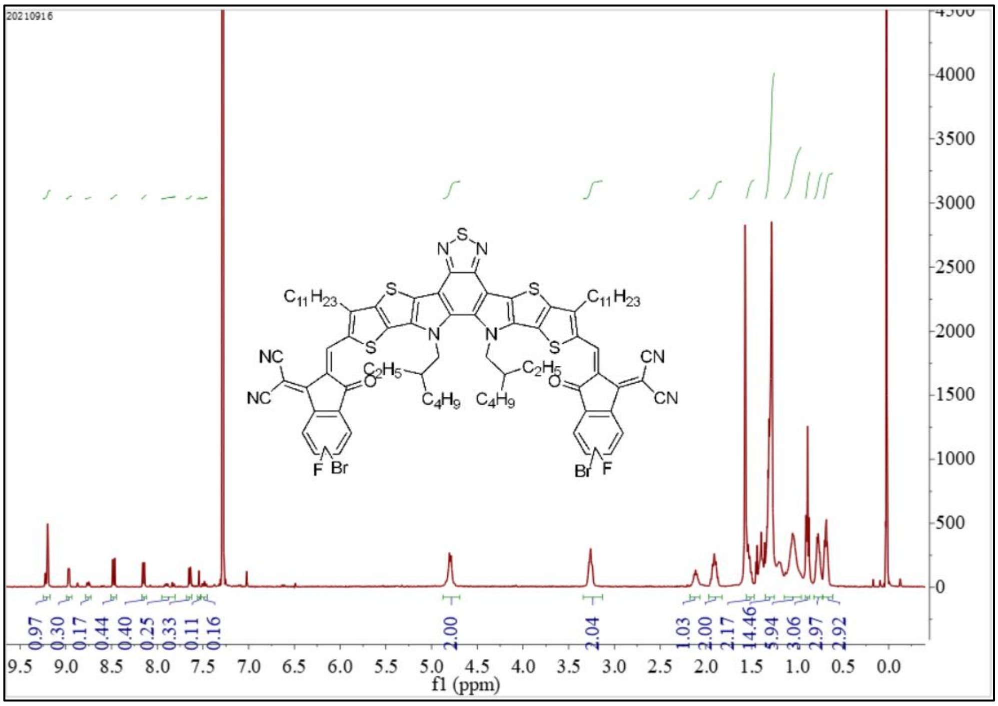

Supplementary Figure 40. ^{1}H NMR spectrum of **compound 33(**FBr-Y6). MS (MADIL-TOF) m/z: $[M]^+$: calcd for $C_{82}H_{86}Br_2F_2N_8O_2S_5$; found $[M+1]^+$:1571.428.

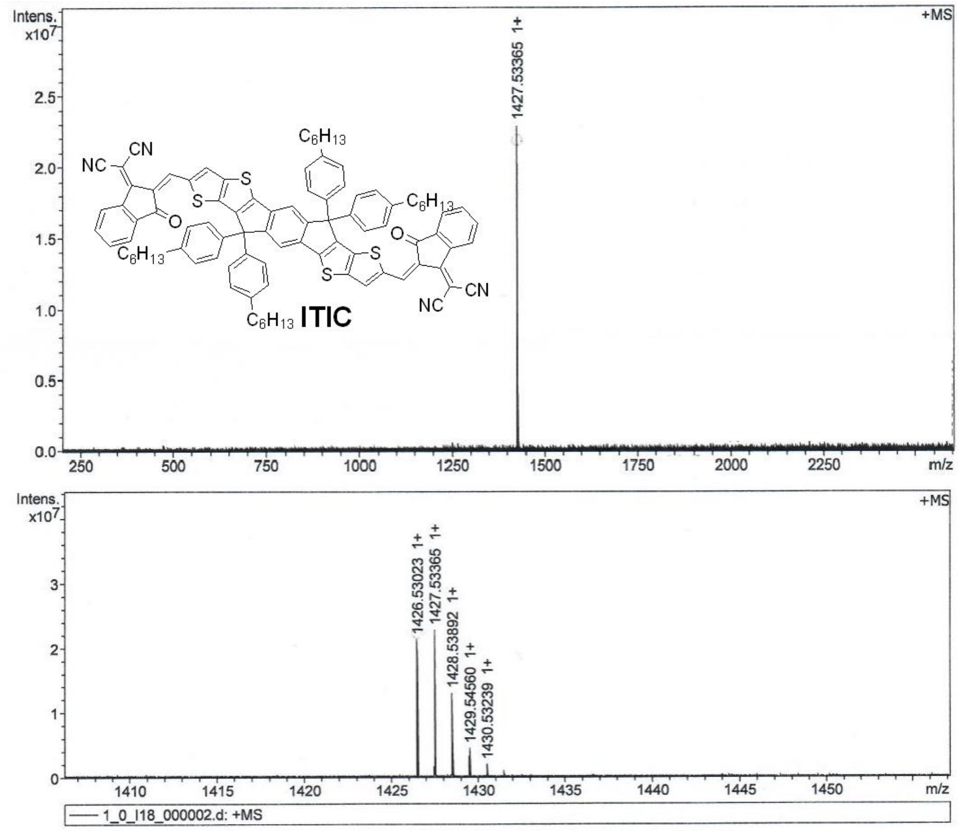

Meas. m/z	#	Ion Formula	Score	m/z	err [ppm]	Mean err [ppm]	mSigma	rdb	e⁻ Conf	N-Rule
1426.530231	1	C94H82N4O2S4	100.00	1426.531513	-0.9	0.1	139.9	56.0	odd	ok

Supplementary Figure 41. High resolution mass spectra of ITIC

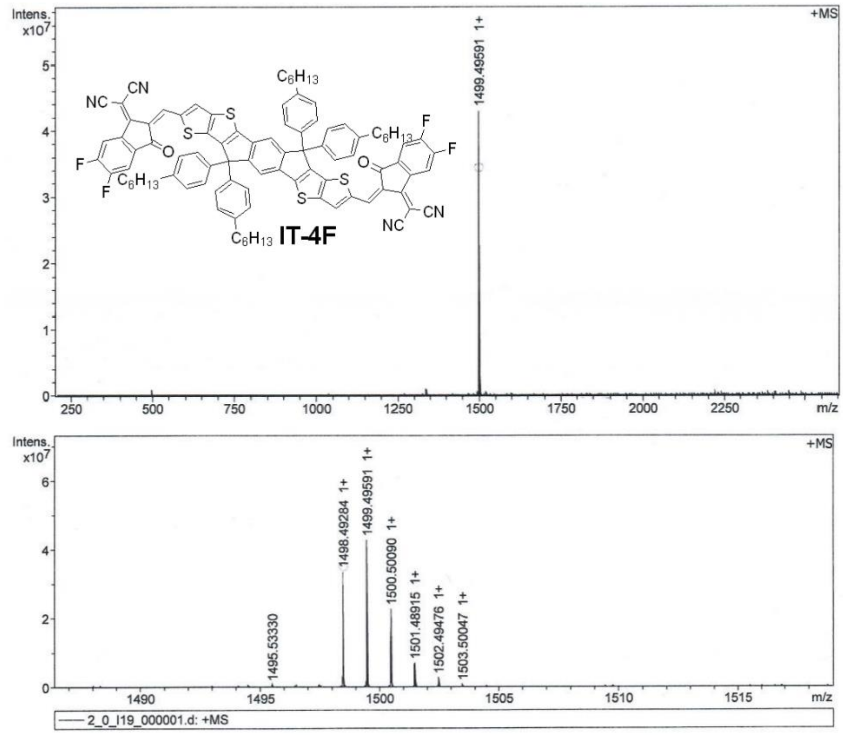

Meas. m/z	#	Ion Formula	Score	m/z	err [ppm]	Mean err [ppm]	mSigma	rdb	e⁻ Conf	N-Rule
1498.492839	1	C94H78F4N4O2S4	100.00	1498.493826	0.7	0.1	156.7	56.0	odd	ok

Supplementary Figure 42. High resolution mass spectra of IT-4F

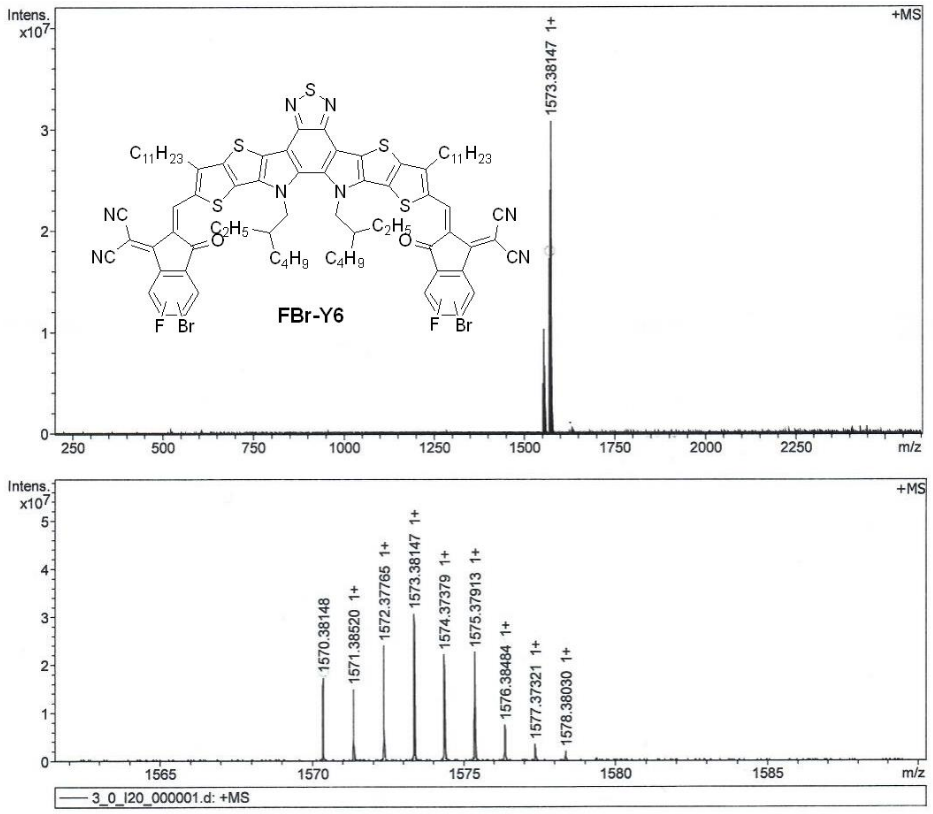

Meas. m/z	#	Ion Formula	Score	m/z	err [ppm]	Mean err [ppm]	mSigma	rdb	e⁻ Conf	N-Rule
1570.381485	1	C82H86Br2F2N8O2S5	100.00	1570.380661	0.5	0.8	131.9	42.0	odd	ok

Supplementary Figure 43. High resolution mass spectra of FBr-Y6

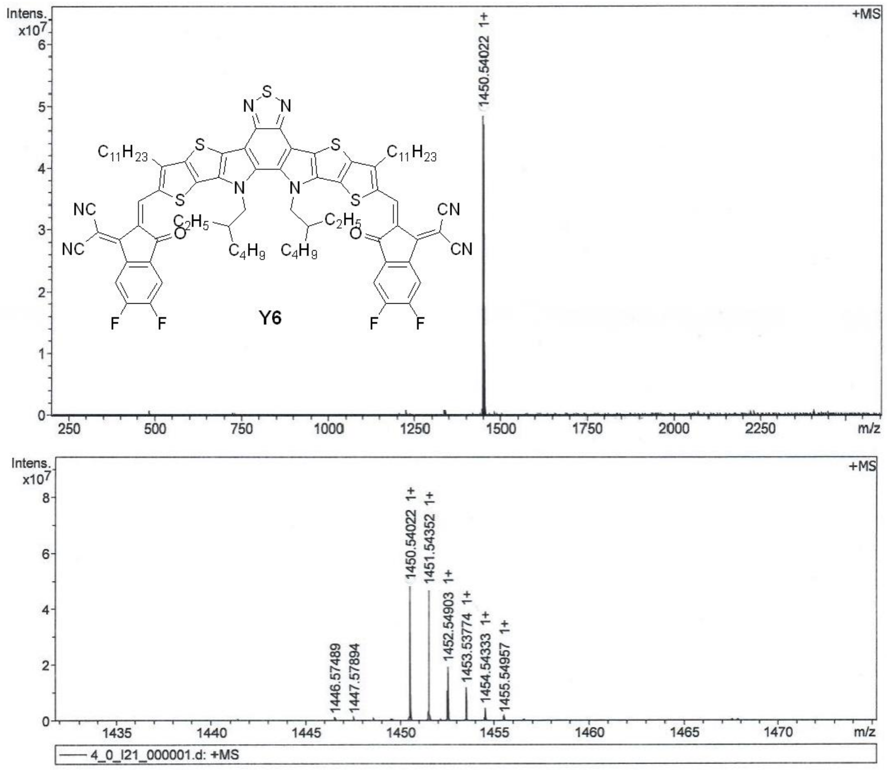

Meas. m/z	#	Ion Formula	Score	m/z	err [ppm]	Mean err [ppm]	mSigma	rdb	e⁻ Conf	N-Rule
1450.540224	1	C82H86F4N8O2S5	100.00	1450.540793	0.4	0.1	116.2	42.0	odd	ok

Supplementary Figure 44. High resolution mass spectra of Y6

Supplementary Tables

Supplementary Table 1. Screening of solutions using $BF_3 \cdot OEt_2$ or $GaCl_3$ as catalyst.[a]

Entry	1	2	3	4	5	6	7	8	9	10	11
Lewis acid	cyclohexane	DCM [e]	Xylene [d]	CCl_4 [d]	toluene [d]	chlorobenzene [d]	DMF	DMSO	THF	CH_3CN	Pyridine
Yield [b] (%)	0	64	>99%	55	>99%	18	0	0	0	0	0
Yield [c] (%)	0	80	>99%	60	>99%	30	0	0	0	0	0

[a] Performed with ITIC-CHO (0.1 mmol), and IC (0.2 mmol) in 5 ml solution and Acetic anhydride (0.1 mL). [b] With the adding of $GaCl_3$ (5 mol %). [c] With the adding of $BF_3 \cdot OEt_2$ (5 equiv. relative to ITIC-CHO). [d] Yield determined by ^1H NMR. [e] Isolated Yield.

Supplementary Table 2. Screening of different anhydrides with $GaCl_3$ (5 mol %) as catalyst [a].

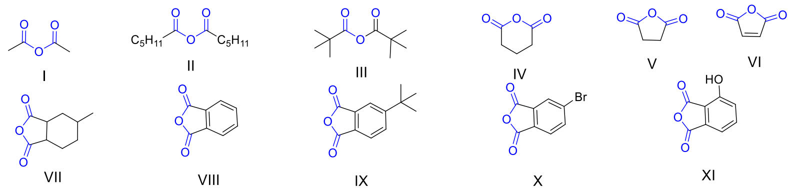

Entry	Anhydrides	*T* (°C)	Time (min)	Yield [b] (%)
1	I	60	30	>99%
2	II	60	40	>99%
3	III	60	60	>99%
4	IV	60	150	>99%
5	V	60	360	0
6	V	90	360	22
7	VI	60	360	0
8	VI	90	240	>99%
9	VII	60	360	0
10	VII	90	180	100
11	VIII	60	360	0
12	VIII	90	300	25
13	IX	60	360	0
14	IX	90	300	31
15	X	60	360	0
16	X	90	360	12
17	XI	60	360	0
18	XI	90	360	0

[a] Performed with ITIC-CHO (0.1 mmol), IC (0.2 mmol), anhydrides (10 mol %) in toluene (5 ml). [b] Determined by ^1H NMR.

Regarding the cyclic anhydrides, glutaric anhydride (IV) led to full completion of the reaction within 150 min (entry 4), while succinic anhydride (V) resulted in a low conversion yield even at higher temperature (entries 5-6), probably due to its ring strain. Notably, partial release of such ring strain, through increasing its conjugation (VI) or connection with cyclohexane outside the ring (VII), could promote a quantitative conversion (entries 7-10) at increased temperature (90 °C). As for the aromatic carboxylic anhydrides (VIII-XI), they were found to be less effective due to their low solubility in toluene (entries 11-18). The efforts in adding an alkyl chain (IX) or bromine atom (VII) proved to be fruitless, with only 31% and 12% yields for the desired product at higher temperature. The ineffectiveness of anhydride k was likely related with its phenolic hydroxy group, which would destroy the interaction of the catalyst with the aldehyde (entry 18).

Supplementary Table 3. Screening of different anhydrides with $BF_3{\cdot}OEt_2$ (5 equiv relative to ITIC-CHO) [a].

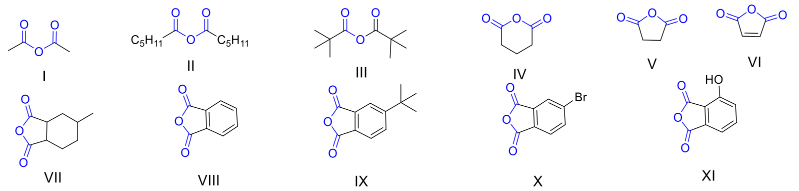

**Entry**	Anhydrides	*T* (°C)	Time (min)	Yield [b] (%)
1	I	25	30	>99%
2	II	25	40	>99%
3	III	25	60	>99%
4	IV	60	360	15
5	V	60	360	0
6	VI	60	360	0
7	VII	60	360	0
8	VIII	60	360	0
9	IX	60	360	0
10	X	60	360	0
11	XI	60	360	0

[a] Performed with ITIC-CHO (0.1 mmol), IC (0.2 mmol), anhydrides (10 e.q) in toluene (5 ml). [b] Determined by ^{1}H NMR.

Supplementary Table 4. The yields of SMAs that reported and described in this paper. It can be seen that the yields can be significantly increased with the new method.

compound	Yield[a] (%)	Yield[b] (%)	References
1	94%	N. A.	
2	91%	N. A.	
3	92%	N. A.	
4 (IDIC)	93%	82%	Adv. Mater. 2018, 30, 1705209
5	93%	N. A.	
6	91%	N. A.	
7	90%	N. A.	
8	93%	N. A.	
9	92%	N. A.	
10 (ITIC)	92%	45%	Chem. Mater. 2017, 29, 10294−10298
11(IT-4F)	91%	80%	J. Am. Chem. Soc. 2017, 139, 7148−7151
12	90%	69%	CN 110606856 A
13	90%	N. A.	
14	93%	N. A.	
15	91%	N. A.	
16	92%	N. A.	
17	91%	N. A.	
18	94%	N. A.	
19	91%	N. A.	
20	93%	N. A.	
21	91%	N. A.	
22 (Y5-2BO)	90%	82%	Adv. Energy Mater. 2021,11,2003367.
23(Y6)	95%	64%	Joule, 2018, 3, 1140-1151
24 (BTIC-BO-4C)	92%	53%	J. Mater. Chem. A, 2020, 8, 8903-8912
25 (BTP-4F-P2EH)	95%	82%	Adv. Energy Mater. 2021, 11, 2102596
26	96%	N. A.	
27 (L8-BO)	93%	64%	Nat. Energy 6, 605–613 (2021)
28 (Y5-I-C20-Br)	95%	84%	Adv. Mater. 2020, 32, 2005942
29 (BTP-eC9)	95%	74%	Nat. Commun. 10, 2515 (2019).
30	95%	N. A.	
31	91%	N. A.	
32 (Y5-O-C20-Br)	94%	84%	Adv. Mater. 2020, 32, 2005942
33 (FBr-Y6)	95%	N. A.	

[a] Yield of this work. [b] Yield of reference.

Supplementary Table 5. The calculated chemical synthesis cost for Y6 with a C_g value of 384.2 \$/g. [a]

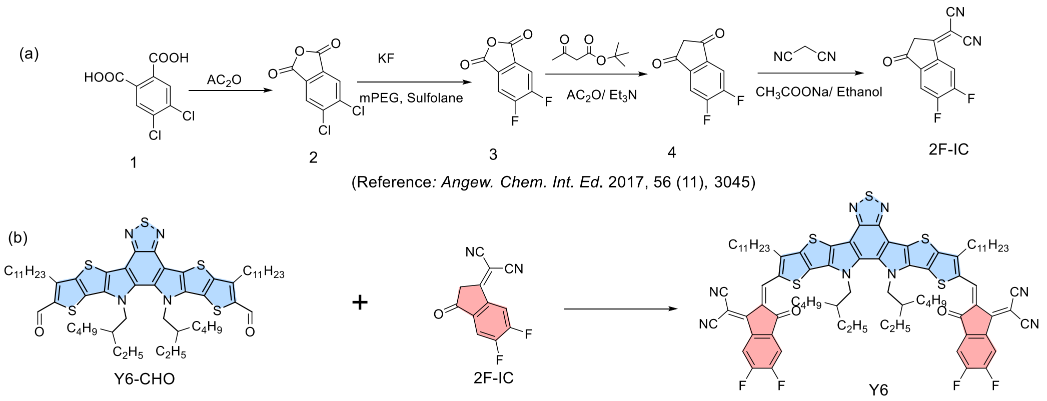

Method	Chemicals	Quantity (g)	Price (RMB/g) [b]	Yields	Price (\$/g)
This method	Y6-CHO	1.00 g	3200	1.39 g (98%)	410.11
	2F-IC	0.45 g	1060		
Conventional method [c]	Y6-CHO	0.15 g	3200	0.14 g (64%)	778.05
	2F-IC	0.21 g	1060		

[a] For conventional method, large volumes of eluent (petroether/DCM) are needed for the tedious column separation, here this factor was not considered for simplify.
[b] Data are from SunaTech Inc.
[c] The data was from previous report (*Joule* 2019, 3, 1140-1151).

Supplementary Table 6. The calculated chemical synthesis cost for IT-4F. [a]

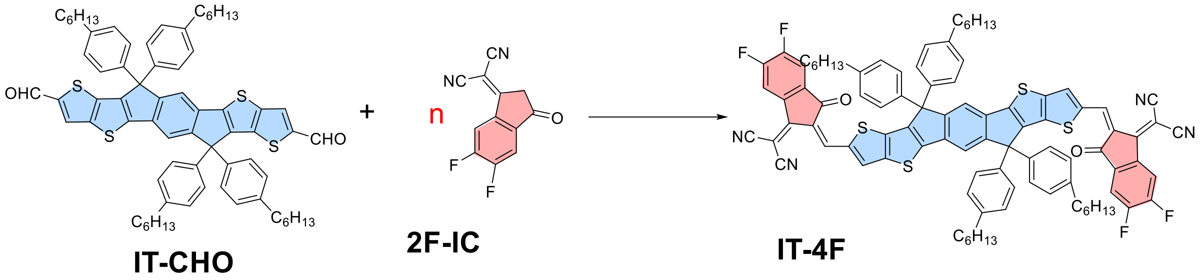

Method	Chemicals	Quantity (g)	Price (RMB/g) [b]	Yields	Price (\$/g)
This method	IT-CHO	1.07 g	1250	1.47 g (98%)	192.30
	2F-IC	0.46 g	1060		
Conventional method [c]	IT-4F-CHO	0.107 g	1250	0.12 g (80%)	330.00
	2F-IC	0.115 g	1060		

[a] For conventional method, large volumes of eluent (petroether/DCM) are needed for the tedious column separation, here this factor was not considered for simplify.
[b] Data are from SunaTech Inc.
[c] The data was from previous report (*J. Am. Chem. Soc.* 2017, 139, 7148−7151).

Supplementary Table 7. The calculated chemical synthesis cost for ITIC. [a]

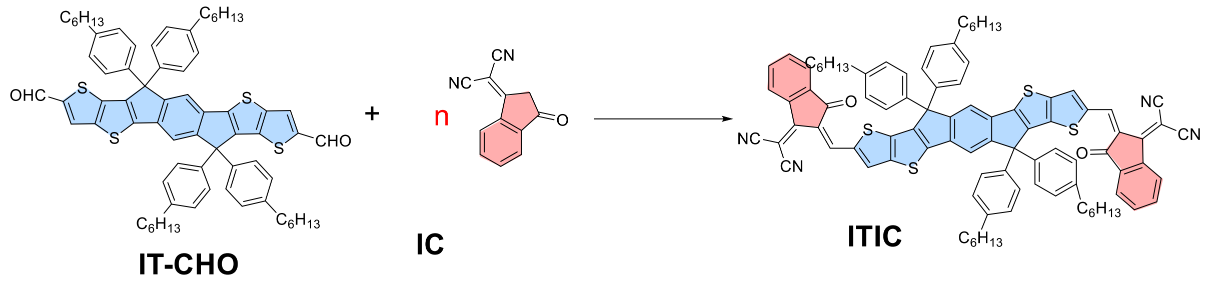

Method	Chemicals	Quantity (g)	Price (RMB/g) [b]	Yields	Price ($/g)
This method	IT-CHO	1.07 g	1250	1.40 g (98%)	164.80
	IC	0.39 g	390		
Conventional method [c]	IT-4F-CHO	0.107 g	1250	0.08 g (54%)	302.66
	IC	0.058 g	390		

[a] For conventional method, large volumes of eluent (petroether/DCM) are needed for the tedious column separation, here this factor was not considered for simplify.
[b] Data are from SunaTech Inc.
[c] The data was from previous report (*Chem. Mater*. 2017, 29, 10294−10298).

Supplementary Table 8. Photovoltaic parameters of the four OPV cells under the AM 1.5 G, 100 mW cm^{-2}.[a]

D: A	V_{OC} [V]	FF [%]	J_{SC} [mA cm^{-2}]	PCE [%]	IPCE [mA cm^{-2}]
PM6: Y6	0.845 (0.844 ± 0.001)	77.00 (76.94 ± 0.24)	26.37 (25.82 ± 0.42)	17.15 (16.76 ± 0.24)	25.98
	0.752 ± 0.001 [b]	44.15 ± 0.02 [b]	19.744 ± 0.04 [b]	6.34 ± 0.01 [b]	
PM6: FBr-Y6	0.840 (0.836 ± 0.003)	70.23 (69.72 ± 0.77)	25.58 (22.83 ± 0.077)	15.09 (14.90 ± 0.19)	24.85
PM6: IT-4F	0.878 (0.864 ± 0.008)	72.90 (71.50 ± 1.10)	20.43 (20.49 ± 0.038)	13.08 (12.65 ± 0.28)	19.10
PBDB-T: ITIC	0.889 (0.889 ± 0.001)	71.90 (71.15 ± 0.90)	16.68 (16.37 ± 0.100)	10.66 (10.36 ± 0.16)	15.92

[a] average parameters are calculated from 5 independent cells. [b] $GaCl_3$ (0.5%, wt%) was added.
