# Supplementary material for: Low-cost synthesis of small molecule acceptors makes polymer solar cells commercially viable
Source: Nat Commun. 2022 Jun 27;13:3687. doi: 10.1038/s41467-022-31389-y (PMC9237043; doi:10.1038/s41467-022-31389-y)
Supplement: Supplementary file 2 — Description of Additional Supplementary Files [file 41467_2022_31389_MOESM2_ESM.docx]

**Description of Additional Supplementary Files**

File Name: Supplementary Movie 1

Description: The synthesis of IT-4F (**10**, in Figure 3d) via the BF_3_∙OEt_2_-catalyzed Knoevenagel condensation.
