# Supplementary material for: Low-cost synthesis of small molecule acceptors makes polymer solar cells commercially viable
Source: Nat Commun. 2022 Jun 27;13:3687. doi: 10.1038/s41467-022-31389-y (PMC9237043; doi:10.1038/s41467-022-31389-y)
Supplement: Supplementary file 4 — Solar Cells Reporting Summary [file 41467_2022_31389_MOESM4_ESM.pdf]

nature research

Corresponding author(s): Zhi-Guo Zhang

Solar Cells Reporting Summary

Nature Research wishes to improve the reproducibility of the work that we publish. This form is intended for publication with all accepted papers reporting the characterization of photovoltaic devices and provides structure for consistency and transparency in reporting. Some list items might not apply to an individual manuscript, but all fields must be completed for clarity.

For further information on Nature Research policies, including our data availability policy, see Authors & Referees.

ü Experimental design

Please check: are the following details reported in the manuscript?

1. Dimensions

Item	Yes	No	Details
Area of the tested solar cells	☒	☐	Area of the tested solar cells is provided in main text, section"Device fabrication and characterization of the OSCs"
Method used to determine the device area	☒	☐	Area of the tested solar cells is provided in main text, section"Device fabrication and characterization of the OSCs"

2. Current-voltage characterization

Item	Yes	No	Details
Current density-voltage (J-V) plots in both forward and backward direction	☐	☒	Only the plots in forward direction was supplied.
Voltage scan conditions<br>*For instance: scan direction, speed, dwell times*	☒	☐	Area of the tested solar cells is provided in main text, section"Device fabrication and characterization of the OSCs"
Test environment<br>*For instance: characterization temperature, in air or in glove box*	☒	☐	Area of the tested solar cells is provided in main text, section"Device fabrication and characterization of the OSCs"
Protocol for preconditioning of the device before its characterization	☐	☒	No preconditioning protocol.
Stability of the J-V characteristic<br>*Verified with time evolution of the maximum power point or with the photocurrent at maximum power point; see ref. 7 for details.*	☐	☒	This work focus on synthesis method for SMAs.

3. Hysteresis or any other unusual behaviour

Item	Yes	No	Details
Description of the unusual behaviour observed during the characterization	☐	☒	No hysteresis was observed in our device.
Related experimental data	☐	☒	No.

4. Efficiency

Item	Yes	No	Details
External quantum efficiency (EQE) or incident photons to current efficiency (IPCE)	☒	☐	IPCE curve was provided in Figure 3c
A comparison between the integrated response under the standard reference spectrum and the response measure under the simulator	☒	☐	Relative information is provided Figure 3
For tandem solar cells, the bias illumination and bias voltage used for each subcell	☐	☒	Our cells were single solar cells.

5. Calibration

Item	Yes	No	Details
Light source and reference cell or sensor used for the characterization	☒	☐	Area of the tested solar cells is provided in main text, section"Device fabrication and characterization of the OSCs"
Confirmation that the reference cell was calibrated and certified	☒	☐	Area of the tested solar cells is provided in main text, section"Device fabrication and characterization of the OSCs"

Calculation of spectral mismatch between the reference cell and the devices under test	☐ Yes ☒ No	Spectral mismatch factor was not considered.

6. Mask/aperture

Size of the mask/aperture used during testing	☐ Yes ☒ No	We didn't use masks during testing in the lab.
Variation of the measured short-circuit current density with the mask/aperture area	☒ Yes ☐ No	The masked test results are provided

7. Performance certification

Identity of the independent certification laboratory that confirmed the photovoltaic performance	☐ Yes ☒ No	*Explain why this information is not reported/not relevant.*
A copy of any certificate(s) *Provide in Supplementary Information*	☐ Yes ☒ No	*Explain why this information is not reported/not relevant.*

8. Statistics

Number of solar cells tested	☒ Yes ☐ No	Number of solar cells tested is provided in Supplementary Table 8
Statistical analysis of the device performance	☒ Yes ☐ No	Statistical results of the devices are listed in Supplementary Table 8.

9. Long-term stability analysis

Type of analysis, bias conditions and environmental conditions *For instance: illumination type, temperature, atmosphere humidity, encapsulation method, preconditioning temperature*	☐ Yes ☒ No	*Explain why this information is not reported/not relevant.*
